# Bleeding Risk with Long-Term Low-Dose Aspirin: A Systematic Review of Observational Studies

**DOI:** 10.1371/journal.pone.0160046

**Published:** 2016-08-04

**Authors:** Luis A. García Rodríguez, Mar Martín-Pérez, Charles H. Hennekens, Peter M. Rothwell, Angel Lanas

**Affiliations:** 1 Spanish Centre for Pharmacoepidemiologic Research (CEIFE), Madrid, Spain; 2 Charles E. Schmidt College of Medicine, Florida Atlantic University, Boca Raton, Florida, United States of America; 3 Stroke Prevention Research Unit, Nuffield Department of Clinical Neurosciences, John Radcliffe Hospital, University of Oxford, Oxford, United Kingdom; 4 University of Zaragoza, University Clinic Hospital, IIS Aragón, CIBERehd, Zaragoza, Spain; University of Colorado Denver, UNITED STATES

## Abstract

**Background:**

Low-dose aspirin has proven effectiveness in secondary and primary prevention of cardiovascular events, but is also associated with an increased risk of major bleeding events. For primary prevention, this absolute risk must be carefully weighed against the benefits of aspirin; such assessments are currently limited by a lack of data from general populations.

**Methods:**

Systematic searches of Medline and Embase were conducted to identify observational studies published between 1946 and 4 March 2015 that reported the risks of gastrointestinal (GI) bleeding or intracranial hemorrhage (ICH) with long-term, low-dose aspirin (75–325 mg/day). Pooled estimates of the relative risk (RR) for bleeding events with aspirin versus non-use were calculated using random-effects models, based on reported estimates of RR (including odds ratios, hazard ratios, incidence rate ratios and standardized incidence ratios) in 39 articles.

**Findings:**

The incidence of GI bleeding with low-dose aspirin was 0.48–3.64 cases per 1000 person-years, and the overall pooled estimate of the RR with low-dose aspirin was 1.4 (95% confidence interval [CI]: 1.2–1.7). For upper and lower GI bleeding, the RRs with low-dose aspirin were 2.3 (2.0–2.6) and 1.8 (1.1–3.0), respectively. Neither aspirin dose nor duration of use had consistent effects on RRs for upper GI bleeding. The estimated RR for ICH with low-dose aspirin was 1.4 (1.2–1.7) overall. Aspirin was associated with increased bleeding risks when combined with non-steroidal anti-inflammatory drugs, clopidogrel and selective serotonin reuptake inhibitors compared with monotherapy. By contrast, concomitant use of proton pump inhibitors decreased upper GI bleeding risks relative to aspirin monotherapy.

**Conclusions:**

The risks of major bleeding with low-dose aspirin in real-world settings are of a similar magnitude to those reported in randomized trials. These data will help inform clinical judgements regarding the use of low-dose aspirin in prevention of cardiovascular events.

## Introduction

Aspirin (acetylsalicylic acid; ASA) is one of the world’s most widely used drugs [1], with substantial clinical evidence demonstrating its analgesic, antipyretic, and anti-inflammatory properties [2]. Aspirin also exhibits antiplatelet activity by irreversibly inhibiting production of the eicosanoid thromboxane A_2_ (TXA_2_), a powerful promoter of platelet aggregation [3, 4]. This property underlies the effectiveness of aspirin in the prevention of occlusive cardiovascular (CV) events, including myocardial infarction [5, 6], stroke [7, 8], and transient ischaemic attack [9, 10], as demonstrated in randomized trials of primary and secondary prevention. More recently, post-hoc analyses of randomized trials have shown that aspirin reduces the incidence and mortality of colorectal cancer. Observational studies have also suggested possible benefits on other cancers [11]. The use of low-dose aspirin is, however, associated with several adverse effects, the most clinically relevant of which are major extracranial bleeding events, specifically GI bleeding [12]. In addition, aspirin increases the risk of the serious but rare event, intracranial (including intracerebral) hemorrhage (ICH) [13].

In the secondary prevention of cardiovascular disease (CVD), the absolute benefits of aspirin far outweigh the absolute risks of major bleeding events [14]. In primary prevention, however, the net benefit of aspirin is smaller than for secondary prevention [6]. Thus, recent clinical guidelines recommend that for primary prevention, clinicians assess the balance between the risk of occlusive CV events and the risk of major bleeding events on an individual basis [14–17].

In addition to establishing the level of bleeding risk associated with low-dose aspirin use, there is a need to identify the factors that influence the risk of bleeding with aspirin therapy and the magnitudes of these effects on the risk. For example, many patients will be taking concomitant medications that have been shown to increase the risk of GI bleeding when taken alone, including anticoagulants, other antiplatelet agents, and non-steroidal anti-inflammatory drugs (NSAIDs) [18], while other patients may have a history of peptic ulcer, which is known to increase the risk of upper GI bleeding (UGIB) considerably [19].

Data from both randomized controlled trials (RCTs) and observational studies are required to assess the risks associated with low-dose aspirin, given the notable differences between these study types in the populations included, levels of monitoring, and, therefore, reported outcomes. Indeed, a large proportion of patients who take aspirin in the real world (such as elderly patients, patients with a history of ulcers or GI complaints, and those who are also taking other gastrotoxic drugs) are excluded from some RCTs, and there is a lack of robust data from real life clinical practice. A recent systematic review of RCTs and observational studies investigated the bleeding risk with aspirin therapy, and age and the presence of *Helicobacter pylori* were identified as factors that may increase the risk of GI bleeding events in individuals taking aspirin; however, this review included only a small number of observational studies, all of which were conducted in the UK [20].

In the present systematic review, data from a large number of observational studies, conducted across multiple countries, were assessed in order to determine the risks of the most clinically relevant adverse effect, GI bleeding, and the serious but rare event, ICH, in patients taking low-dose aspirin in real-world settings. The influence of risk factors, including age and concomitant medications, on the association between low-dose aspirin and bleeding events was also assessed.

## Methods

### Search Strategy

Systematic searches of Medline and Embase were performed for terms relating to epidemiology, aspirin, and aspirin safety (specifically GI bleeding and ICH) in the titles and abstracts of papers published between 1946 and 4 March 2015 (see S1 File for details of the search strategy. No published protocol was followed). The search was restricted to studies conducted in humans and to publications written in English. Reviews, editorials, comments, clinical trials and pediatric studies were excluded, as were studies using only aspirin doses higher than 325 mg per day. The results were supplemented by a PubMed search for publications only available online that were not identified by Medline or Embase. In addition, one relevant article published after completion of the searches was included.

**Fig 1:**
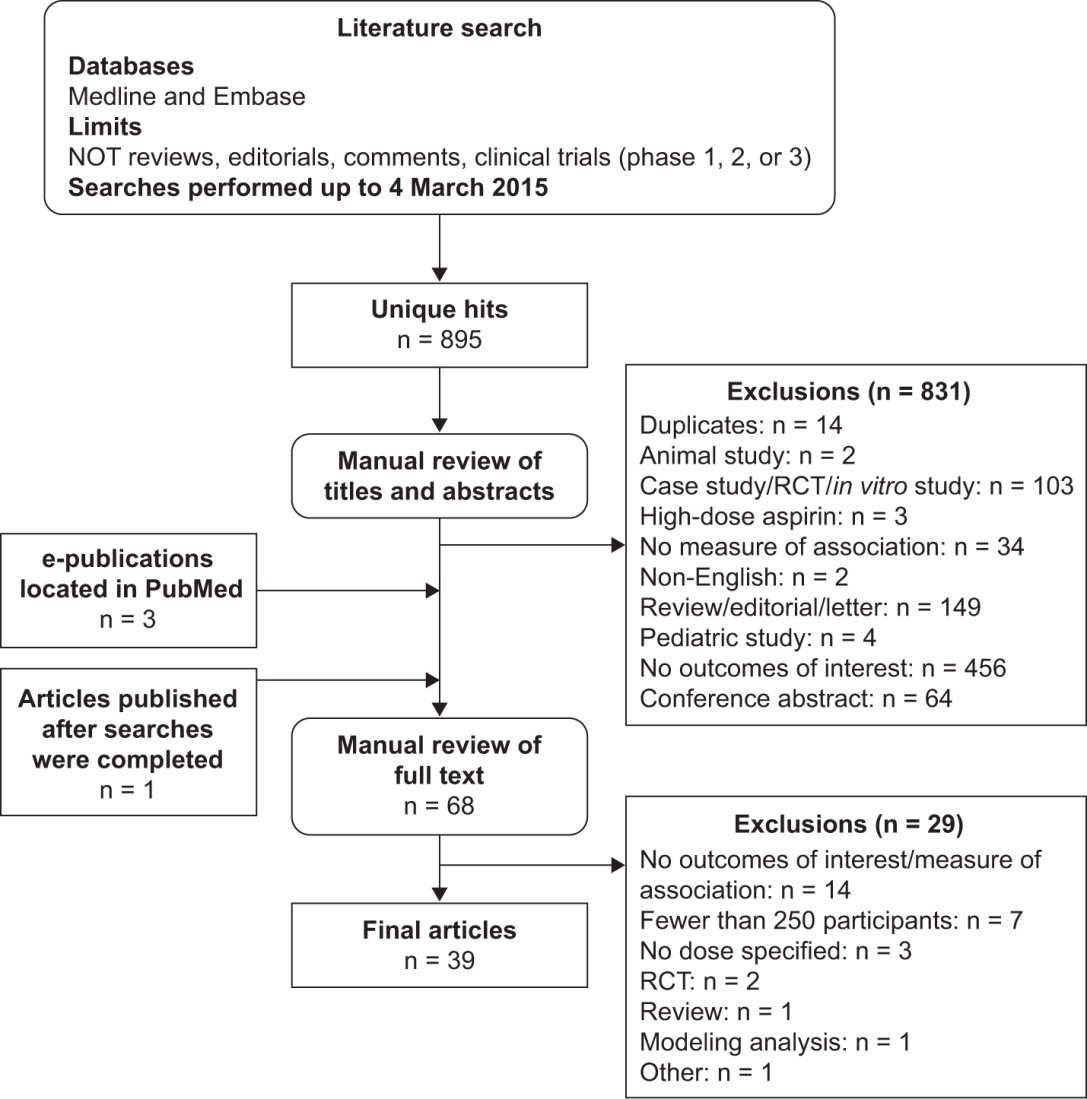
Flow diagram of the systematic literature search. RCT, randomized controlled trial.

After removal of duplicates, the identified references were manually screened on the basis of titles and abstracts. Observational studies identified as containing potentially relevant information were subsequently reviewed as full-text articles. Articles were included only if they reported measures of association (odds ratio [OR], relative risk [RR], hazard ratio [HR], incidence rate ratio [IRR], or standardized incidence ratio [SIR]) between aspirin use and the risk of major bleeding events, specifically events reported as GI bleeding or ICH; a full list of exclusion criteria is detailed in S2 File. A flow chart of the systematic literature search is shown in Fig 1.

### Data Extraction

Details of study design, study population, data source, aspirin dose(s), and indication were extracted from the articles. If available, information on the frequency and duration of aspirin use was also retrieved. The outcomes recorded were the incidences of GI bleeding and ICH and measures of their association (OR, RR, HR, IRR, SIR) with low-dose aspirin (75–325 mg per day). In addition, several factors were examined for their potential predictive effects on the risk of bleeding, such as age, history of peptic ulcer, and *H*. *pylori* infection; when available, the incidences of GI bleeding and ICH and the measures of association for these factors were recorded. The effects of medications taken concomitantly with low-dose aspirin (including proton pump inhibitors [PPIs], clopidogrel, and non-steroidal anti-inflammatory drugs [NSAIDs]) on the risk of bleeding were also documented.

### Statistical Analysis

Pooled estimates of the RRs for GI bleeding and ICH with low-dose aspirin versus non-use were calculated by random-effects models. This model weights individual studies by sample size and variance (both within- and between-study variance) and yields a pooled point estimate and 95% confidence interval (CI). Heterogeneity was assessed using the *I*^2^ statistic. Pooled estimates of the RR with low-dose aspirin in case–control and cohort studies were compared; pooled estimates of the overall RR of low-dose aspirin in all studies were also calculated. All statistical analyses were performed using STATA 12 (Stata Corp, College Station, TX).

## Results

### Summary of Studies Included

A total of 39 articles met the inclusion criteria for the review (S1 Table). Among these observational studies, there were 23 case–control studies, including one case–crossover study [21], and 16 cohort studies, one of which was a long-term, post-trial follow-up of a randomized trial [22]. Notably, some of the case–control studies identified were nested in well-defined cohorts [23–29].

Twenty-three studies reported data from European countries (seven each from Spain and the UK), nine from the USA, and seven from Asia (S1 Table). All except five studies were published since the beginning of 2000 and almost half (18 studies) were published since the start of 2010. When specified, the duration of aspirin use ranged from 1 month to more than 20 years, and reported follow-up was 4–14 years. Studies evaluated daily and/or regular use of aspirin. The criteria for participants to be considered ‘current users’ of aspirin varied among studies: the maximum time period between stopping aspirin use and the study index date ranged from 7 to 90 days.

### Risk of Gastrointestinal Bleeding with Low-Dose Aspirin

#### All gastrointestinal bleeding

Eight studies reported measures of the RR for all GI bleeding events, with low-dose aspirin compared with non-use: two case–control studies [18, 30], one case–crossover study [21], and five cohort studies (S1 Table) [22, 31–34]. Two studies showed no increase in the risk of GI bleeding with low-dose aspirin [22, 31], whereas six studies showed a significant increase compared with non-use [18, 21, 30, 32–34]. The highest risk was reported in Japanese patients prescribed low-dose aspirin for CVD for more than 1 year, although the confidence intervals were wide [33]. With the exception of this study, the RRs were similar in cohort and case–control studies. The range of the estimates of the RRs for GI bleeding with low-dose aspirin was 0.99–4.64, with most studies reporting values between 0.99 and 1.6.

**Fig 2:**
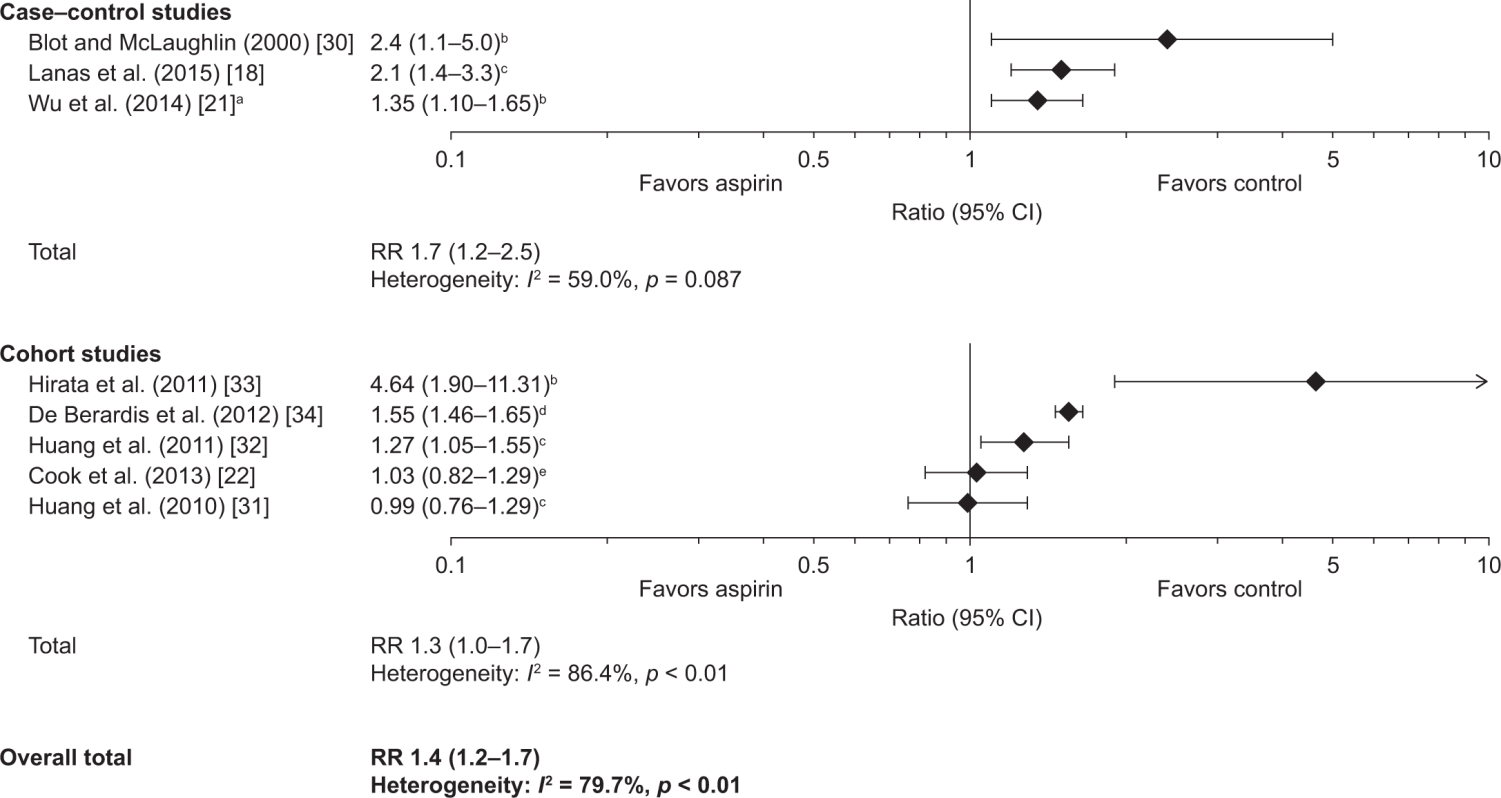
Risk of gastrointestinal bleeding with low-dose aspirin. Data are shown as adjusted OR, adjusted RR (unless otherwise stated), adjusted IRR, or multivariate HR, plus 95% CIs, for the risk of any gastrointestinal bleeding with low-dose aspirin vs no aspirin. A test for heterogeneity (*I*^*2*^ statistic) is provided. ^a^Case-crossover study; ^b^adjusted OR; ^c^adjusted RR; ^d^adjusted IRR; ^e^multivariate HR. CI, confidence interval; HR, hazard ratio; IRR, incidence rate ratio; OR, odds ratio; RR, relative risk.

The pooled estimates of the RR for GI bleeding with low-dose aspirin were 1.7 (95% CI: 1.2–2.5) for case–control studies and 1.3 (95% CI: 1.0–1.7) for cohort studies (Fig 2). There was significant heterogeneity among cohort studies (*I*^2^ = 86.4%) and less heterogeneity among case–control studies (*I*^2^ = 59.0%). The overall pooled estimate of the RR for GI bleeding with low-dose aspirin, for all eight studies, was 1.4 (95% CI: 1.2–1.7) (Fig 2), although there was significant heterogeneity among studies (*I*^2^ = 79.7%).

**Fig 3:**
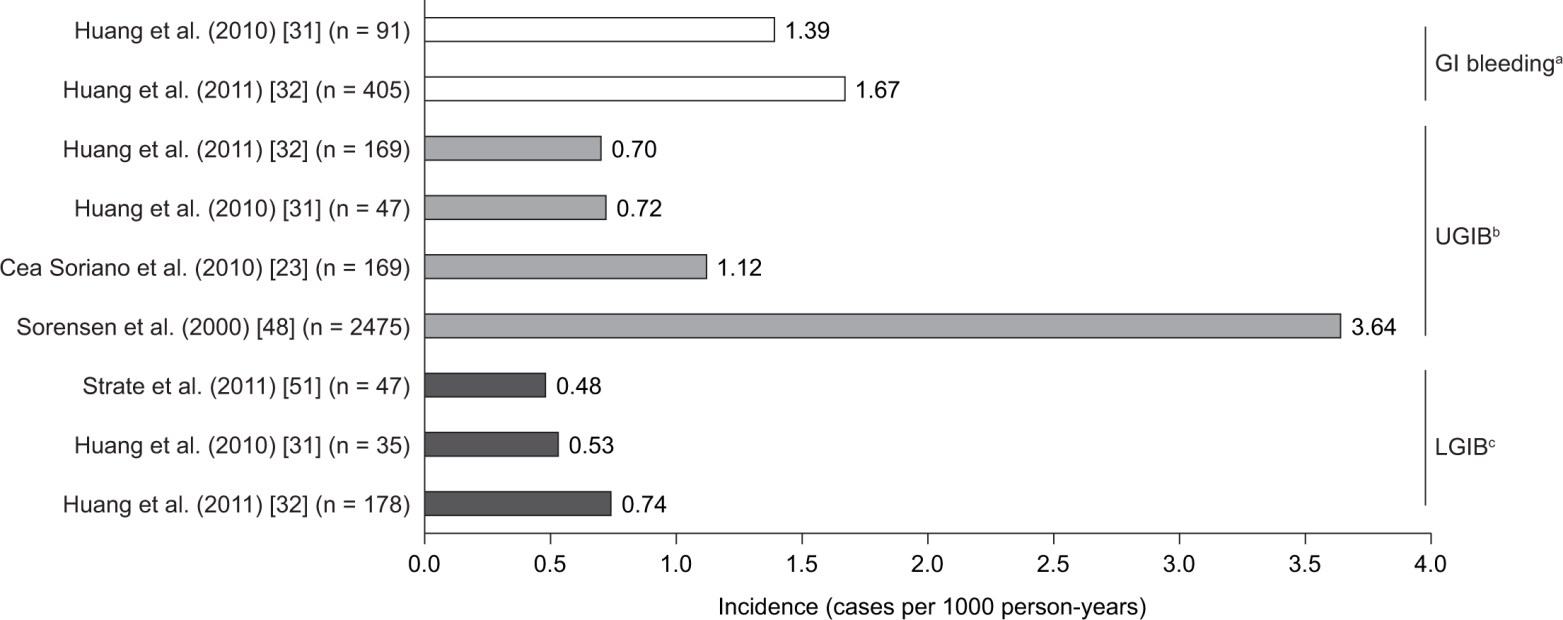
Incidence (cases per 1000 person-years) of gastrointestinal bleeding with low-dose aspirin. ^a^Incidences for all GI bleeding as reported in the original studies, without specification of location within the tract; ^b^incidences specifically for upper GI bleeding as reported in the original studies; ^c^incidences specifically for lower GI bleeding as reported in the original studies. GI, gastrointestinal; LGIB, lower gastrointestinal bleeding; UGIB, upper gastrointestinal bleeding.

The overall incidence (as cases per 1000 person-years) of GI bleeding with low-dose aspirin were reported in two cohort studies conducted in the USA, one of which involved only men (1.39 events per 1000 person-years) and the other of which involved only women (1.67 events per 1000 person-years) [31, 32] (Fig 3).

#### Upper gastrointestinal bleeding

**Fig 4:**
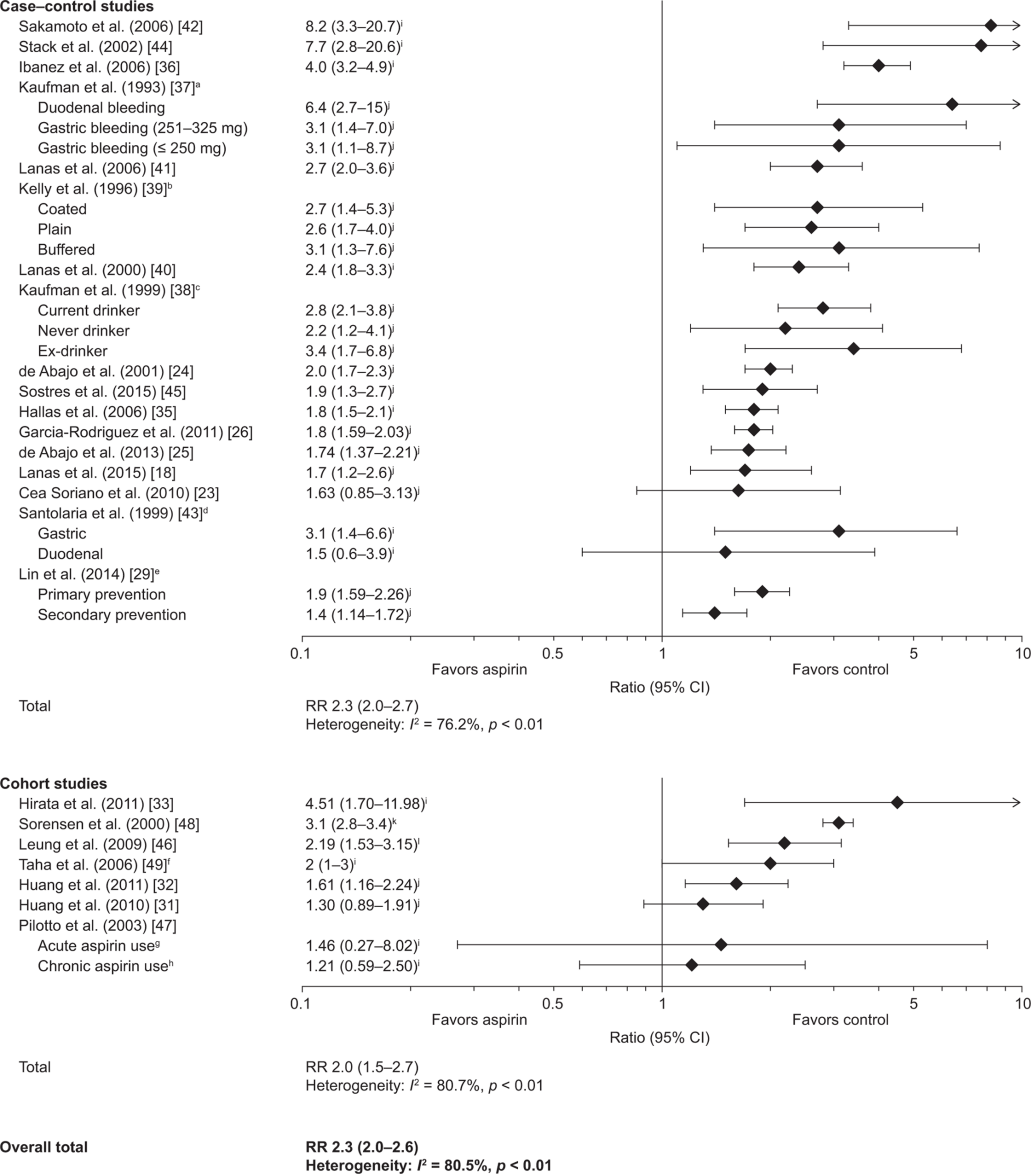
Risk of upper gastrointestinal bleeding with low-dose aspirin: case–control studies and cohort studies. For case–control studies, data are shown as adjusted or multivariate ORs or RRs, plus 95% CIs, for the risk of upper gastrointestinal bleeding with low-dose aspirin vs no aspirin. For cohort studies, data are shown as multivariate ORs, SIR, multivariate HR, or multivariate RRs, plus 95% CIs, for the risk of upper gastrointestinal bleeding with low-dose aspirin vs no aspirin. A test for heterogeneity (*I*^*2*^ statistic) is provided. ^a^Data for duodenal bleeding and gastric bleeding (defined as bleeds located in the upper gastrointestinal tract, at sites other than the duodenum) [≤ 250 and 251–325 mg per day]; ^b^Data for plain, enteric-coated, and buffered aspirin; ^c^Data for different alcohol consumption categories (current drinker/never drinker/ex-drinker); ^d^Data for gastric ulcer and duodenal ulcer bleeding (defined as the presence of melena, haematemesis or haematochezia with a substantial decrease in haemoglobin and a peptic ulcer identified endoscopically); ^e^Data for primary and secondary prevention; ^f^Risk of erosive esophagitis; ^g^Acute defined as regular or sporadic aspirin use for 5–30 days before endoscopy; ^h^Chronic defined as regular aspirin use for more than 1 month before endoscopy; ^i^OR; ^j^RR; ^k^SIR; ^l^HR. CI, confidence interval; HR, hazard ratio; OR, odds ratio; RR, relative risk; SIR, standardized incidence ratio.

Most studies reporting GI bleeding evaluated the effects of low-dose aspirin on UGIB. Of the 24 studies, 17 were case–control studies [18, 23–26, 29, 35–45] and seven were cohort studies [31–33, 46–49]. Low-dose aspirin was associated with a significant increase in the risk of UGIB compared with non-use in most studies. In the case–control studies, the majority of RRs for UGIB with low-dose aspirin were in the range 1.4–4.0 and the pooled estimate was 2.3 (95% CI: 2.0–2.7) (Fig 4). RRs in the cohort studies fell within a similar range (1.2–4.5), with a pooled estimate of 2.0 (95% CI: 1.5–2.7). The overall pooled estimate of the RR for UGIB with low-dose aspirin, for all 24 studies, was 2.3 (95% CI: 2.0–2.6). There was significant heterogeneity among both case–control (*I*^2^ = 76.2%) and cohort studies (*I*^2^ = 80.7%) and among all studies (*I*^2^ = 80.5%). Three case–control studies reported risks higher than those in the other studies, but these three studies all involved relatively few cases of UGIB (n < 300) and controls, and the CIs were wide [37, 42, 44].

One study compared the RR for UGIB with low-dose aspirin in the primary and secondary prevention of CVD [29]. The RR for UGIB with low-dose aspirin alone compared with non-use was higher in the primary than in the secondary prevention cohort (adjusted RR [95% CI]: 1.90 [1.59–2.26] and 1.40 [1.14–1.72], respectively). However, the baseline absolute risk of UGIB was higher in the secondary than in the primary prevention cohort; patients in the secondary cohort were older and were more likely to have a history of ulcers and to use concomitant medications, such as NSAIDs, clopidogrel, and oral anticoagulants. Indeed, the analysis showed that the absolute increase in risk of UGIB with low-dose aspirin was higher in the secondary prevention cohort than in the primary prevention cohort.

**Fig 5:**
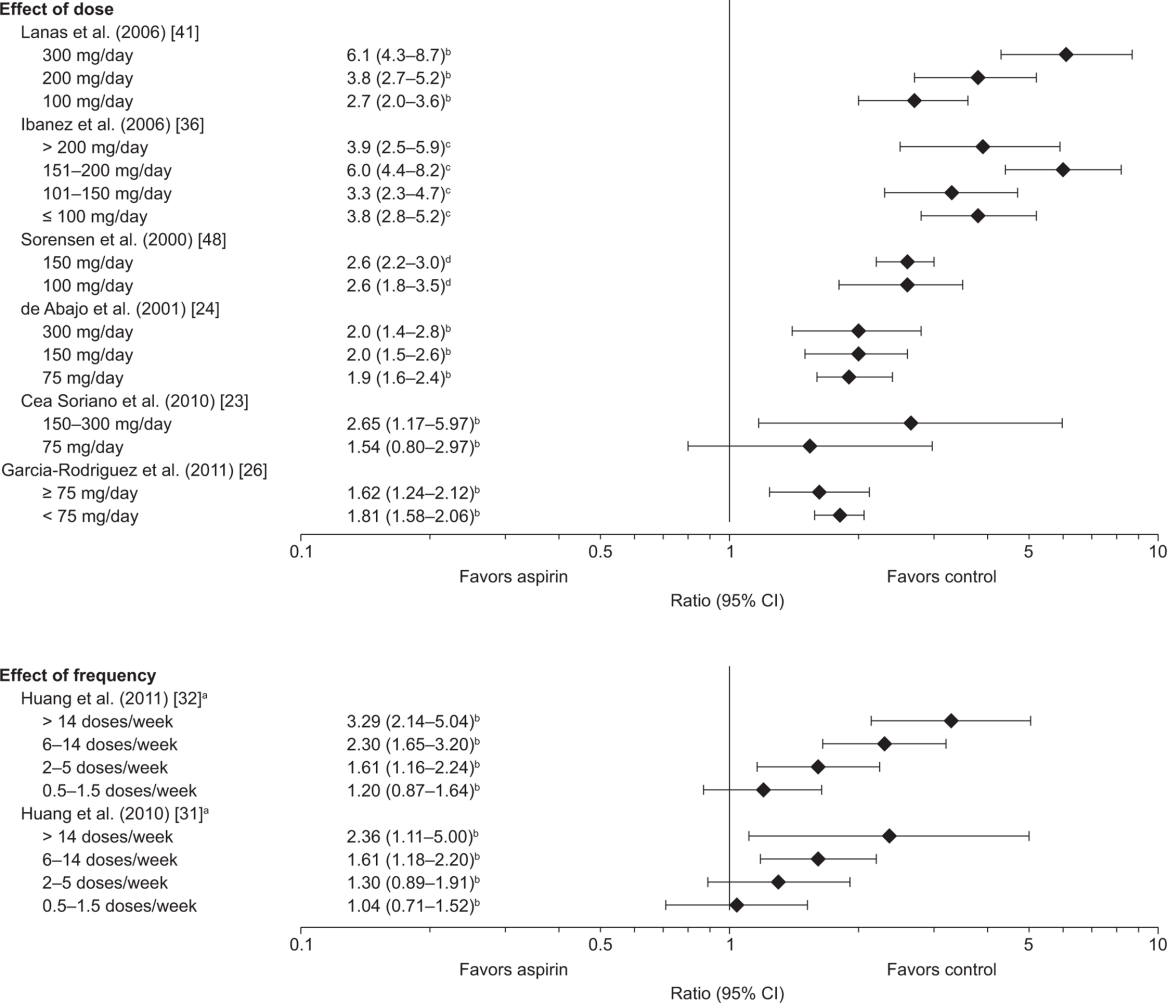
Risk of upper gastrointestinal bleeding with low-dose aspirin: effects of dose and frequency of use. Data are shown as adjusted RR, adjusted OR, or SIR, plus 95% CIs, for the risk of upper gastrointestinal bleeding with low-dose aspirin vs no aspirin. ^a^One dose equals one 325 mg tablet; ^b^adjusted RR; ^c^Adjusted OR; ^d^SIR. CI, confidence interval; OR, odds ratio; RR, relative risk; SIR standardized incidence ratio.

Eight studies evaluated the effects of aspirin dose or dosing regimen on the risk of UGIB (Fig 5) [23, 24, 26, 31, 32, 36, 41, 48]. Two studies reported an increase in the risk of UGIB with increasing frequency of doses [31, 32] and a third demonstrated a clear dose-dependency for UGIB with low-dose aspirin in a Spanish case–control study [41]. Other studies reported little difference in RRs for different low-dose aspirin regimens [24, 26, 36, 48]. RRs for some doses (e.g. 300 mg) showed marked differences between the studies.

Two studies suggested that the duration of continuous low-dose aspirin treatment up to 10 or 20 years has little effect on the RR of UGIB, when adjusted for treatment dose [31, 32]. Similarly, another study reported an increase in the RR of UGIB with low-dose aspirin monotherapy, irrespective of whether patients had received treatment for less than or more than 1 year [26]. However, it has also been shown that the risk of UGIB with low-dose aspirin is greatest during the first 2 months of therapy, and decreases with increasing treatment duration [24], with a similar effect also being observed in another study [41].

The effects of recent or past use or discontinuation of low-dose aspirin, compared with current use, on the risk of UGIB were assessed in five studies [23, 24, 26, 35, 41]. All five studies showed a lower risk of UGIB with recent or past use compared with current use of low-dose aspirin. One study reported recent discontinuation of low-dose aspirin (i.e. use ending 15–180 days before the index date) in 11.2% of cases and 13.1% of controls [23]. The data suggested that the risk of UGIB was reduced in those who had recently discontinued low-dose aspirin when compared with patients who continued treatment (adjusted RR: 0.71 [95% CI: 0.42–1.20]). Among the patients who had discontinued, the risk of UGIB was higher for those who had discontinued aspirin for safety-related reasons (RR compared with continued use: 2.18 [95% CI: 0.80–5.95]) than for patients who had discontinued for non-safety related reasons, primarily non-adherence (RR: 0.52 [95% CI: 0.28–0.99]).

Four studies reported overall incidences of UGIB with low-dose aspirin, which were in the range of 0.70–3.64 cases per 1000 person-years (Fig 3). Incidences were similar in studies conducted in the USA [31, 32] and the UK [23] and higher in a Danish cohort study [48].

#### Lower gastrointestinal bleeding

Six studies evaluated the risk of LGIB associated with the use of low-dose aspirin compared with non-use: two case–control studies [18, 50] and four cohort studies [31–33, 51]. RRs for LGIB with low-dose aspirin varied from 0.8 to 5.0 (S1 Fig), with a pooled estimate of 1.8 (95% CI: 1.1–3.0). There was significant heterogeneity among studies (*I*^2^ = 81.1%). The highest risk was reported in a Japanese study involving patients with CVD prescribed low-dose aspirin for more than 1 year (OR 5.03 [95% CI: 0.59–43.16]) [33]; however, few (n < 10) cases of bleeding were observed, so the CI was wide and the finding not statistically significant. A second Japanese study reported a significantly increased risk of LGIB with low-dose (100 mg) aspirin (OR 3.7 [95% CI: 1.3–10.9]), although again, relatively few cases of bleeding (n = 44) were observed [50]. In a recent Spanish case–control study (> 1000 observed cases of bleeding), low-dose aspirin was associated with a 2.7-fold increased risk of LGIB (including bleeds in both the small and large bowel) compared with non-use [18].

Two cohort studies involving men enrolled in the Health Professionals Follow-up Study examined the effects of aspirin on LGIB [31, 51]. One reported a significant increase in the risk of diverticular bleeding with low-dose aspirin (2–5.9 tablets of 325 mg per week; multivariate HR: 2.32 [95% CI: 1.34–4.02]) compared with no aspirin [51]. However, no significant increase in risk was observed with either 0.1–1.9 or ≥ 6 aspirin tablets per week (multivariate HR 1.58 [95% CI: 0.88–2.82] and 1.65 [95% CI: 0.84–3.26], respectively). The other cohort study reported no significant increase in risk of LGIB with 2–5 tablets per week or any of the other doses studied (0.5–1.5, 6–14, or > 14 tablets per week) [31]. Similarly, none of these aspirin doses was associated with a significant increase in risk of LGIB in women enrolled in the Nurses’ Health Study [32].The duration of continuous aspirin use also had no significant effect on risk of LGIB in either of these studies [31, 32].

Three studies reported overall incidence of LGIB among patients receiving low-dose aspirin (Fig 3); the incidences were similar across the studies (0.48–0.74 cases per 1000 person-years) [31, 32, 51].

### Risk of Intracranial Hemorrhage with Low-Dose Aspirin

**Fig 6:**
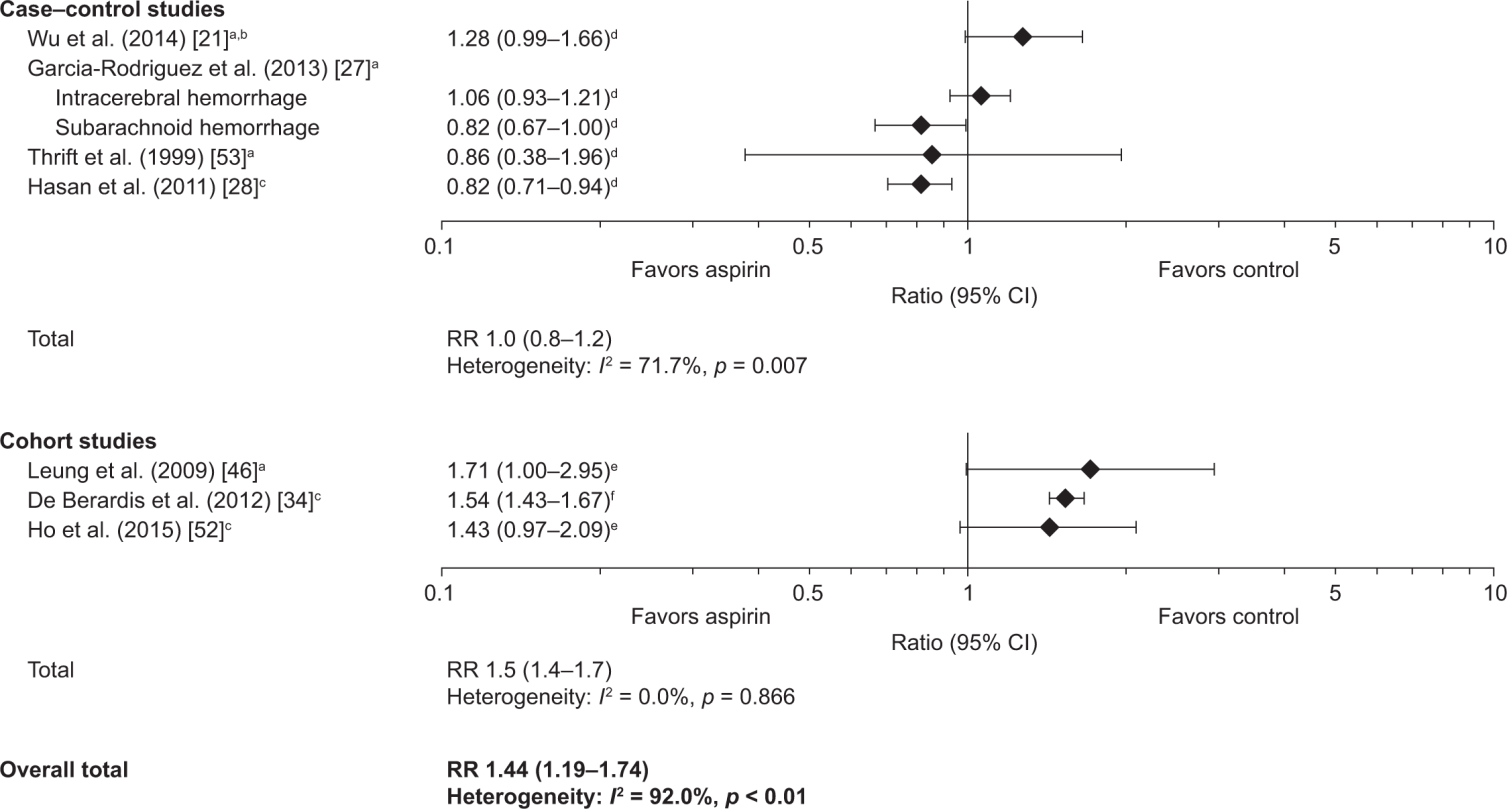
Risk of intracranial (including intracerebral) hemorrhage with low-dose aspirin. Data are shown as adjusted OR, multivariate HR or adjusted IRR, plus 95% CIs, for the risk of intracranial (including intracerebral) hemorrhage with low-dose aspirin vs no aspirin. A test for heterogeneity (*I*^*2*^ statistic) is provided. ^a^Intracerebral hemorrhage/hemorrhagic stroke; ^b^Intracranial hemorrhage; ^c^Case-crossover study; ^d^adjusted OR; ^e^multivariate HR; ^f^adjusted IRR. CI, confidence interval; HR, hazard ratio; ICH, intracerebral hemorrhage; IRR, incidence rate ratio; OR, odds ratio; SAH, subarachnoid hemorrhage.

Seven studies reported the risk of ICH associated with low-dose aspirin therapy: three cohort studies [34, 46, 52], three case–control studies [27, 28, 53], and one case-crossover study [21]. In most studies, low-dose aspirin was not associated with a significantly increased risk of ICH (Fig 6). Estimates of RRs for ICH with low-dose aspirin were 0.82–1.71 across the studies, and for most studies the 95% CI included 1.0. The pooled estimates of the RR for ICH with low-dose aspirin were 1.0 (95% CI: 0.8–1.2) for case–control studies and 1.5 (95% CI: 1.4–1.7) for cohort studies, and 1.4 (95% CI 1.2–1.7) overall. Heterogeneity was high among all studies (*I*^2^ = 92.0%) and among case–control studies (*I*^2^ = 71.7%), but there was less heterogeneity among cohort studies (*I*^2^ < 0.05%). One study reported the overall incidence of ICH with low-dose aspirin (8.0 cases per 1000 person-years) in a cohort of patients with non-valvular atrial fibrillation [52].

### Potential Predictive Factors

#### Age

Four studies reported increasing incidences of major bleeding events (including all GI bleeding and ICH [34] and UGIB [23, 25, 48]) with increasing age (S2 Fig). The incidence of UGIB was similar across the three studies that reported this outcome and was generally higher in men than in women. The magnitude of the increase in UGIB incidence with age was more pronounced in two studies than in the third study. One of these two studies showed an increase of 3.24 cases per 1000 person-years in men aged ≥ 70 years compared with men aged 16–59 years [48], and the other demonstrated an increase of 2.22 cases per 1000 person-years in men aged ≥ 80 years compared with men aged 40–59 years [25].

Data on the effects of age on the RR of major bleeding events with low-dose aspirin were available from eight studies: two assessing all major bleeding events (GI bleeding and ICH) [21, 34], two evaluating major GI bleeding [31, 32], three reporting data for UGIB [24, 29, 48], and one assessing ICH [53]. There was no clear evidence that the RR of bleeding with low-dose aspirin increases with increasing age (S3 and S4 Figs).

#### Peptic ulcer and *Helicobacter pylori* infection

Both a history of peptic ulcer and *H*. *pylori* infection were associated with a significant increase in the risk of UGIB (S2 Table) [35, 42–45, 54]. However, the combination of low-dose aspirin use and *H*.*Pylori* infection was not associated with a significant increase in the risk of duodenal or gastric ulcer bleeding compared with either variable alone in one study [43]. In another study, there was no increase in the risk of UGIB beyond the sum of the independent effects of the two variables [45].

#### Concomitant medications

Some studies evaluated the effects of PPIs on the risk of major bleeding events when taken in combination with aspirin (S3 Table)[18, 23, 34, 36, 40, 47]. In one study, the combination of PPI plus low-dose aspirin was not associated with an increased risk of UGIB compared with non-use of both agents, in contrast to the use of low-dose aspirin alone [18]. In addition, another study showed that omeprazole plus low-dose aspirin was associated with a reduced risk of UGIB compared with patients taking low-dose aspirin alone [40].

Several studies suggested that the use of low-dose aspirin in combination with other NSAIDs leads to an increased risk of bleeding events compared with use of low-dose aspirin alone (S4 Table) [23, 26, 40]. One study reported a substantially increased risk of upper gastrointestinal complications (bleeding or perforation) with aspirin and high-dose NSAID compared with non-use of both drugs beyond the sum of their independent effects, but no such interaction was observed with aspirin and low/medium-dose NSAIDs. The NSAIDs assessed in this study included ibuprofen, diclofenac, and mefenamic acid [24].

A higher risk of UGIB with low-dose aspirin plus clopidogrel compared with low-dose aspirin alone has also been demonstrated (S4 Table) [23, 26, 35]. Clopidogrel plus low-dose aspirin was not associated with an increased risk of intracerebral hemorrhage compared with non-use of both agents [27]. In addition, when low-dose aspirin was used in combination with other anticoagulant medications, the risk of major bleeding was increased compared with non-use or the monotherapies (S4 Table) [24, 27, 34, 35].

One study reported a small, but not significant, increase in the risk of UGIB with selective serotonin reuptake inhibitor (SSRI) use compared with non-use in patients not receiving aspirin [26] (S5 Table). One study reported a greater number of hospitalizations for UGIB with current use of SSRIs compared with the expected number for non-SSRI users (observed to expected ratio: 3.6), and the risk of hospitalization was increased further with SSRI plus low-dose aspirin (S5 Table) [55]. Another study, however, showed no significant difference in the incidence of major bleeding events between SSRI users and individuals receiving both low-dose aspirin and an SSRI [34].

## Discussion

This systematic review of 39 observational studies provides an indication of the magnitudes of the risk of major bleeding events associated with long-term, low-dose aspirin therapy in the real world.

### Effects of Low-Dose Aspirin on the Risk of Major Bleeding Events

The incidence of all GI bleeding events with low-dose aspirin varied between 0.5 and 3.6 cases per 1000 person-years. The lower end of this range concurs with results from the 2009 meta-analysis of RCTs by the Antithrombotic Trialists’ Collaboration (ATTC), which calculated an incidence of 0.5 cases of major extracranial bleeding events per 1000 person-years [6]. A recent review estimated the incidence of all GI bleeding events in the UK population, using results from trials included in the ATTC analysis, as well as those from a small number of UK observational studies [20]. The estimated range of 0.79–4.92 cases per 1000 person-years in men aged from 50–84 years is comparable to the range of incidence values presented in this review.

The majority of studies reported an increased RR of GI bleeding with low-dose aspirin compared with non-use, which is consistent with findings from previous meta-analyses of RCTs [6]. The pooled estimate of the RR for all GI bleeding (presumably from the whole GI tract) with low-dose aspirin was 1.4, with pooled estimates of 1.3 and 1.7 in the cohort and case–control studies, respectively. In the recent analysis of observational studies conducted in the UK, the risk of all GI bleeding events with low-dose aspirin was slightly higher than in this review, with a total risk ratio of 1.88 in cohort studies and a total OR of 2.41 in case–control studies [20]. The same article also provided an overview of RCTs that reported RRs for any GI bleeding events with low-dose aspirin. These RRs were lower than those for observational studies (range 1.31–1.96). Similarly, the 2009 ATTC meta-analysis reported an RR of 1.54 (95% CI: 1.30–1.82) for major extracranial bleeds with low-dose aspirin in six primary prevention trials [6], and a recent systematic review conducted for the US Preventive Services Task Force (USPSTF) reported an OR of 1.59 (95% CI: 1.32–1.91) for major GI bleeding with aspirin (50–500 mg per day) in seven CVD primary prevention trials and an IRR of 1.55 (5.58 and 3.60 events per 1000 person-years in aspirin users and non-users, respectively) for the same outcome in cohort data [56]. Given the general agreement between the data from cohort studies and those from RCTs and the greater susceptibility of some case–control studies to recall bias, the lower estimate of risk derived from the cohort studies and RCTs of less than a twofold increase in GI bleeding with aspirin compared with non-use, seems likely to be the most reliable and is consistent with our findings.

In the present review, the RRs for UGIB with low-dose aspirin were higher than those for all GI bleeding events (ranging from 1.4 to 8.2 for case–control studies and from 1.21 to 4.51 for cohort studies). These findings are as expected given the well-established adverse effects of aspirin on the upper GI mucosa, which include direct cytotoxic effects on the epithelium, as well as impaired platelet aggregation [57].

The association between low-dose aspirin and the risk of bleeding was less strong for LGIB than for UGIB, with wide variation in the data. Notably, data from three studies showed there was a significantly increased risk of bleeding in individuals treated with low-dose aspirin (RRs: 2.3–3.7), and two studies showed a non-significant increase in the risk of LGIB (RRs: 1.13–5.03), compared with non-use. The pooled analysis suggested that low-dose aspirin increases the risk of LGIB by less than twofold. Indeed, there is evidence for aspirin causing mucosal injury in the small bowel as well as in the upper GI tract, in addition to its antiplatelet effect [58, 59]. Thus, more studies are warranted to establish the magnitude of risk of LGIB with low-dose aspirin.

In the case–control studies included in the present review, there was a lack of a significant effect of low-dose aspirin on the risk of ICH. However, the three cohort studies included in this analysis all reported increased relative risks of ICH with aspirin compared with non-use. Thus, the overall pooled estimate of RR for ICH with low-dose aspirin was 1.4, suggesting a trend towards a small increased risk of ICH with aspirin. These findings are consistent with those from the 2009 meta-analysis by the ATTC, which showed a non-significant increase in the incidence of hemorrhagic stroke in patients taking low-dose aspirin for primary prevention (rate ratio: 1.32 [95% CI: 1.00–1.75]) [6] and the recent systematic review performed for the USPSTF, which reported an OR of 1.33 (95% CI: 1.03–1.71) for hemorrhagic stroke with aspirin in CVD primary prevention trials [56]. In considering these findings, it should be borne in mind that, although life-threatening, ICH is a rare event in individuals under the age of 70 years [60].

Only one of the studies that reported risks of ICH provided figures for its incidence, and this study was performed in a cohort of patients with non-valvular atrial fibrillation. As would be expected, the incidence of ICH was very high in both aspirin-treated and untreated groups (8.0 and 5.3 cases per 1000 person-years, respectively). Several recent observational studies have estimated the incidence of ICH in patient groups more representative of the general population. One study calculated an incidence of sub-arachnoid hemorrhage of 0.11 per 1000 person-years in the Danish population [61], while another reported the incidences of intracerebral and subarachnoid hemorrhage in the UK population to be 0.15 and 0.11 cases per 1000 person-years, respectively [60]. Combined data from two earlier population studies estimated an overall ICH incidence of 0.16 per 1000 person-years between 1981 and 2006 in England and Wales [62]. These figures are in agreement with the incidence of hemorrhagic stroke reported in the ATTC meta-analysis of primary prevention studies (0.18 and 0.13 cases per 1000 person-years in aspirin treated and untreated groups, respectively) [6].

### Impact of Aspirin Dose and Duration of Treatment on Bleeding Risk

Two cohort studies included in the present review, which investigated a wide range of aspirin dosing regimens (0.5–>14 tablets (325 mg) per week), showed an increased risk of UGIB with more frequent dosing [31, 32]. Studies investigating a possible correlation between dose and risk of bleeding did not find any consistent effects [23, 24, 26, 36, 41, 48], suggesting that the risk of bleeding associated with the use of aspirin within the defined ‘low-dose’ range may not vary substantially according to dose.

Increasing the duration of aspirin use was not associated with increased RRs of bleeding. Indeed, one study included in this review reported smaller risk estimates when aspirin was used for more than 5 years, compared with short-term aspirin use [24]. Another study reported no significant effect of the duration of aspirin use on the risk of GI bleeding, and it was postulated that long-term aspirin use is associated with mucosal adaptations that protect against bleeding events [32]. These findings, together with the demonstration of increased benefits of aspirin with longer-term use [63], support the hypothesis of a more favourable benefit–risk ratio for longer-term use of aspirin in primary prevention.

### Influence of Patient Factors on the Risk of Bleeding Associated with Low-Dose Aspirin

#### Age

The studies included in the present review showed fairly consistent RRs for GI bleeding with low-dose aspirin compared with non-use in individuals of different ages. However, the incidence of major bleeding events increased with age, as expected; thus, the absolute risk of bleeding in individuals receiving low-dose aspirin increased with age. Indeed, the 2009 meta-analysis of randomized trials by the ATTC identified age as the most important predictor of the risk of bleeding associated with low-dose aspirin, with an approximate doubling of the absolute risk of bleeding with low-dose aspirin for every 10-year increase in age [6]. It is important to note that any increase in the absolute risk of aspirin-associated bleeding with age would have to be balanced against a possible increased absolute benefit of aspirin in older patients [63].

#### *H*. *pylori* infection

The presence of *H*. *pylori* infection has been postulated to increase the risk of peptic ulcer bleeding in individuals taking low-dose aspirin [45]. Although the studies reviewed here showed an association between *H*. *pylori* infection and GI bleeding, patients taking low-dose aspirin who had *H*. *pylori* infection were not generally at significantly increased risk of ulcer bleeding compared with non-infected individuals taking low-dose aspirin or infected individuals not taking aspirin. A recent review proposed that screening for and eradication of *H*. *pylori* infection could substantially decrease aspirin-related GI damage [20]. The ongoing *Helicobacter* Eradication Aspirin Trial (HEAT), which is due to be completed in April 2017, is assessing whether eradication of *H*. *pylori* has the potential to prevent peptic ulcer bleeding in aspirin users [64].

#### Use of concomitant medications

The ability of PPIs to reduce the risk of aspirin-associated GI bleeding has been observed previously [65] and was also evident in the studies reviewed here [66]. The present review also showed that the risk of GI bleeding was increased in individuals taking low-dose aspirin in combination with other NSAIDs, as expected. Some studies directly compared the use of low-dose aspirin plus NSAIDs with low-dose aspirin alone, with calculated RRs for GI bleeding of 2.6–3.8. The observed inter-study variation is most likely to be attributable to differences in the types and doses of NSAIDs used in the studies.

This review also indicated increases in the risk of UGIB with concomitant therapy with low-dose aspirin and clopidogrel compared with low-dose aspirin alone. The use of SSRIs in combination with aspirin was also associated with an increased risk of UGIB compared with aspirin alone, although the number of studies analyzed was small. There was reported to be no increase in risk of ICH with the combination of low-dose aspirin and clopidogrel versus low-dose aspirin alone. However, a separate study reported an increased risk of both intracerebral and subarachnoid haemorrhage with concomitant use of aspirin and dipyridamole compared with aspirin alone. Further studies are needed to help to clarify which drugs at which doses affect the risks of major bleeding events when given with low-dose aspirin therapy.

### Study Strengths and Limitations

A key strength of this review is that it collates data from a large number (n = 39) of observational studies, which reflect the effects of aspirin in the real world. Limitations include the fact that observational studies are more prone to bias from confounding factors than RCTs and the identified between-study heterogeneity, reflecting the differences in design, aspirin treatment regimen, bleeding definitions, outcomes recorded and calculated and the more diverse patient populations. As a result of this, the pooled estimates of bleeding risk contain a level of uncertainty. However, despite these factors, there was agreement between many of the studies and our findings are generally consistent with results from RCTs.

### Future Implications for the Use of Low-Dose Aspirin in Primary Prevention

These findings contribute to the body of evidence regarding aspirin’s relative benefits and risks in primary prevention. Ongoing and future trials will provide further information on the benefit–risk profile of low-dose aspirin in the prevention of CVD events, particularly in the primary prevention of CVD events. The Aspirin to Reduce Risk of Initial Vascular Events (ARRIVE) trial is assessing the benefits and risks of low-dose aspirin for primary prevention in individuals at moderate risk of CV events [67]. Other trials are investigating the benefit–risk profile of aspirin in specific patient groups, including elderly patients (Aspirin in Reducing Events in the Elderly [ASPREE] trial) [68] and individuals with diabetes (A Study of Cardiovascular Events in Diabetes [ASCEND]) [69].

In addition to the efficacy in reducing the risk of CVD events and the potential increase in risk of major bleeding events, the increasing evidence for the chemopreventive effects of aspirin, most notably in colorectal cancers, merits consideration in clinical decision-making regarding the use of low-dose aspirin for primary prevention, particularly given the overlap in the risk factors for CVD and cancer [70]. In a recent recommendation statement, the USPSTF recommends low-dose aspirin for the primary prevention of CVD and colorectal cancer in adults aged 50–59 years who have a 10-year CVD risk of ≥ 10%, are not at increased risk of bleeding, have a life expectancy of at least 10 years, and are willing to take low-dose aspirin daily for at least 10 years [71]. For adults aged 60–69 years with a 10-year CVD risk of >10%, the USPSTF recommends that the decision to use low-dose aspirin to prevent CVD and colorectal cancer be made on an individual basis, after consideration of the balance between benefits and risks [71]. Data such as those presented here will inform such decision-making regarding the benefit–risk profile of aspirin.

### Conclusions

There was an approximately 40% increased risk of all GI bleeding with low-dose aspirin in the observational studies reviewed here, a finding very similar to that reported in randomized trials [6, 20]. When UGIB was studied separately, there was an approximately twofold increased risk of bleeding with low-dose aspirin. Neither aspirin dose nor the duration of treatment had consistent effects on the RR for GI bleeding. The overall risk of ICH was also increased by approximately 40% with long-term low-dose aspirin, which is also similar to the estimates from randomized trials, although an increase in risk was not consistently reported in all studies. In users of low-dose aspirin, the absolute risk of bleeding, but not the RR for bleeding compared with non-use, increased with age.

Ongoing and future studies will provide further information on the benefit–risk profile of low-dose aspirin in the prevention of CV events, particularly in primary prevention of CVD. In the meantime, by providing estimates of bleeding risks in a real-world setting, the data presented in this review should assist clinicians in making individual clinical judgments [72] on whether to prescribe low-dose aspirin for the prevention of CVD events.

## Supporting Information

S1 Checklist PRISMA Checklist. (DOC)

S1 Fig Risk of LGIB with low-dose aspirin. Data are shown as OR, adjusted or multivariate RR or adjusted HR, plus 95% CIs, for the risk of lower gastrointestinal bleeding with low-dose aspirin vs no aspirin. A test for heterogeneity (reported as *I*^*2*^ statistics, derived from *X*^*2*^ on 5 degrees of freedom [case–control and cohort studies combined] is provided. ^a^Univariate OR. ^b^RR. ^c^OR. ^d^adjusted HR. CI, confidence interval; d.f., degrees of freedom; HR, hazard ratio; LGIB, lower gastrointestinal bleeding; OR, odds ratio; RR, relative risk.
(EPS)

S2 Fig Effect of age on the incidence of major bleeding events with low-dose aspirin. Incidence values are shown for UGIB, except for De Berardis 2012 (gastrointestinal bleeds and ICH). GI, gastrointestinal; ICH, intracranial (including intracerebral) hemorrhage; UGIB, upper gastrointestinal bleeding.
(EPS)

S3 Fig Effect of age on the risk of major bleeding events (all GI bleeding and ICH) with low-dose aspirin. Data are shown as adjusted IRR, crude or adjusted OR or HR, plus 95% CIs, for the risk of major bleeds with low-dose aspirin vs no aspirin. ^a^Major gastrointestinal bleeds only. ^b^adjusted IRR. ^c^OR. ^d^HR. CI, confidence interval; GI, gastrointestinal; HR, hazard ratio; ICH, intracranial (including intracerebral) hemorrhage; IRR, incidence rate ratio; OR, odds ratio.
(EPS)

S4 Fig Effect of age on the risk of major bleeding events (UGIB) with low-dose aspirin. Data are shown as adjusted RR or SIR, plus 95% CIs, for the risk of UGIB with low-dose aspirin vs no aspirin. ^a^SIR. ^b^adjusted RR. CI, confidence interval; IRR, incidence rate ratio; RR, relative risk; SIR, standardized incidence ratio; UGIB, upper gastrointestinal bleeding.
(EPS)

S1 File Search Strategy. (DOCX)

S2 File Exclusion Criteria Applied to Identified Publication. (DOCX)

S1 Table Summary of study details for selected studies. (DOCX)

S2 Table Effect of *Helicobacter pylori* infection on the risk of UGIB. Values are adjusted or multivariate odds ratios unless otherwise indicated. ^a^Adjusted RR. ^b^NSAIDs included aspirin. CI, confidence interval; D, duodenal ulcer; G, gastric ulcer; UGIB, upper gastrointestinal bleeding.
(DOCX)

S3 Table Effect of proton pump inhibitor use on the risk of major bleeding events. ^a^Incidence rate ratio. CI, confidence interval; LGIB, lower gastrointestinal bleeding; OR, odds ratio; PPI, proton pump inhibitor; RR, relative risk; UGIB, upper gastrointestinal bleeding.
(DOCX)

S4 Table Risk of major bleeding events with concomitant medications: clopidogrel, NSAIDs, and anticoagulants. Values are adjusted or multivariate OR, RR, or HR unless otherwise indicated. ^a^Standardized incidence ratio. ^b^No aspirin, no clopidogrel, no vitamin K antagonist, and no dipyridamole. ^c^Secondary prevention cohort. ^d^Incidence rate ratio. CI, confidence interval; coxib, cyclo-oxygenase 2 inhibitor; HR, hazard ratio; LGIB, lower gastrointestinal bleeding; OR, odds ratio; RR, relative risk; UGIB, upper gastrointestinal bleeding.
(DOCX)

S5 Table Effect of SSRI use on the risk of major bleeding events. ^a^Ratio of observed risk to expected risk. ^b^Incidence rate ratio. CI, confidence interval; coxib, cyclo-oxygenase 2 inhibitor; HR, hazard ratio; LGIB, lower gastrointestinal bleeding; OR, odds ratio; RR, relative risk; SSRI, selective serotonin reuptake inhibitor; UGIB, upper gastrointestinal bleeding.
(DOCX)
