# Supplementary material for: Bleeding Risk with Long-Term Low-Dose Aspirin: A Systematic Review of Observational Studies
Source: PLoS One. 2016 Aug 4;11(8):e0160046. doi: 10.1371/journal.pone.0160046 (PMC4973997; doi:10.1371/journal.pone.0160046)
Supplement: S1 File — (DOCX) [file pone.0160046.s006.docx]

**S1 Text. Search Strategy**

The Medline search was carried out using the search terms below. For the Embase search, the MeSH terms were omitted, in order to limit the number of hits to fewer than 1000.

1. **Epidemiology**

(Epidemiology OR incidence OR odds ratio OR prevalence)[MeSH] OR (epidemiolog* OR incidence OR prevalence OR hazard ratio OR relative risk OR rate ratio OR odds ratio)[Title/Abstract].

1. **Aspirin**

Aspirin[MeSH] OR (aspirin OR ASA OR acetylsalicylic acid OR acetylsalicylate OR 2-acetoxybenzoic acid)[Title/Abstract].

1. **Aspirin Safety**

**GI Bleeds**

(Gastrointestinal OR GI OR gastric OR duodenal OR peptic ulcer OR upper gastrointestinal OR lower gastrointestinal OR upper GI OR lower GI)[Title/Abstract] AND (bleed* OR h?emorrhage*)[Title/Abstract] OR gastrointestinal[MeSH].

OR

**Intracranial Bleeds**

(Bleed* OR h?emorrhage*)[Title/Abstract] AND (intracranial OR intracerebral)[Title/Abstract].

1. **Additional Filters**

Humans[MeSH] AND English[lang].

Searches 1–4 were combined using AND.

1. **Limits**

NOT reviews, editorials, comments OR clinical trials (phase 1, 2 or 3).
