# Supplementary material for: Bleeding Risk with Long-Term Low-Dose Aspirin: A Systematic Review of Observational Studies
Source: PLoS One. 2016 Aug 4;11(8):e0160046. doi: 10.1371/journal.pone.0160046 (PMC4973997; doi:10.1371/journal.pone.0160046)
Supplement: S2 File — (DOCX) [file pone.0160046.s007.docx]

**S2 Text. Exclusion Criteria Applied to Identified Publications**

Only high-dose aspirin used (> 325 mg/day)

No aspirin dose specified

Buffered aspirin used

No outcomes of interest reported

No measure of association reported (e.g. odds ratio/hazard ratio)

Case study

Animal study

*In vitro* study

Randomized controlled trial

Review/editorial/letter

Pediatric study

Non-English text

Duplicate

Conference abstract

Fewer than 250 participants
