# Supplementary material for: Bleeding Risk with Long-Term Low-Dose Aspirin: A Systematic Review of Observational Studies
Source: PLoS One. 2016 Aug 4;11(8):e0160046. doi: 10.1371/journal.pone.0160046 (PMC4973997; doi:10.1371/journal.pone.0160046)
Supplement: S1 Table — (DOCX) [file pone.0160046.s008.docx]

**S1 Table.** **Summary of study details for selected studies**

| Study | Design (country/region) | Data source | Indication | Population (n) | Cohort description | Cases/controls definition | Age (mean ± SD), years | Definition of exposure assessment | Reference group | Aspirin dose | Outcome |
| --- | --- | --- | --- | --- | --- | --- | --- | --- | --- | --- | --- |
| Ahsberg *et al*. (2010) (APT) [1] | Cohort, retrospective (Sweden) | Medical records from a single hospital | Not specified | Total: 252  1984 cohort: 94  1994 cohort: 65  2004 cohort: 93 | Hospitalized patients with a discharge diagnosis of GI bleeding (according to ICD) during 1984, 1994, and 2004 | Patients hospitalized for peptic ulcer bleeds at three different time points | *Median (IQR)*  1984:  70 (54–76)  1994:  74 (64–79)  2004:  77 (65–84) | Not specified | Non-users | ‘Low-dose aspirin’ (not defined) | UGIB |
| Ahsberg *et al*. (2010) [2] | Cohort, retrospective (Sweden) | Medical records from a single hospital | Not specified | Total: 731  UGIB: 440  LGIB: 286 | All adult patients hospitalized with a discharge diagnosis of GI bleeding during 1984, 1994, and 2004 | Adult patients hospitalized with a discharge diagnosis of GI bleeding in the Departments of Surgery and (from 2004) Emergency Conditions at Lund University Hospital | Not specified | Not specified | Non-users or by site of bleed, as indicated | ‘Low-dose aspirin’ (not defined) | GI bleeding |
| Blot and McLaughlin (2000) [3] | Case–control (USA) | Study conducted by the ACG | Analgesia | Cases: 627 Controls: 590 | – | Cases: patients hospitalized for GI-associated bleeding (information collected by a mail survey in 1995)    Controls: endoscopy patients without GI bleeding, matched by site of procedure (upper or lower GI tract) | Cases: 60.1  Controls: 55.0  *Range, %*  (cases/controls) < 50:  29.2/37.6 50–64:  23.9/27.6 ≥ 65:  44.8/33.2 | Recent use: ‘current’ use or ‘within the last week’, not defined further | Non-users: no use during the study period | Three categories: ≤ 324,  325–974 and > 974 mg/day | GI bleeding |
| Cea Soriano *et al*. (2010) [4] | Nested case–control (UK) | THIN 2000–2007 | Secondary prevention of cardiovascular and cerebrovascular events | Cases: 169  Controls: 2,000  (from a cohort of 38,077 patients) | UK primary care patients aged 50–84 years with a first prescription of aspirin for secondary prevention during 2000–2007 | Cases: incident cases of UGIB  Controls: frequency matched by sex, age, and follow-up time | *Range, n (%)* (cases/controls)  < 65:  38 (22.5) /468 (23.4) 65–74:  58 (34.3)/670 (33.5)  ≥ 75:  73 (43.2)/862 (43.1) | Current use: the supply of a prescription for aspirin lasted until the index date or ended within the period of 30 days before index date | Non-users: when supply of the most recent prescription ended more than 365 days before the index date, or there was no recorded use at any time between the start and index dates if that interval was smaller than 365 days | 75–300 mg/day | UGIB |
| Cook *et al*. (2013) [5] | Long-term observational follow-up of RCT (USA) | WHS | Not specified | Aspirin: 16,913 Placebo: 16,769  (based on treatment during trial) | Female health professionals, aged 45 years and older, followed-up in post-trial period | Cases: women with GI bleeding (self-reported diagnosis on annual questionnaires in post-trial years 1, 2, 3, and 5) | *Range, n (%)* (Aspirin/placebo)  45–54:  10,392  (61.4)/10,369 (61.8) 55–64:  5,021  (29.7)/4,930 (29.4) ≥ 65:  1,500  (8.9)/1,470 (8.8) | Not specified | Placebo | 100 mg on alternate days | GI bleeding |
| Dalton *et al*. (2003) [6] | Cohort population-based (Denmark) | HDR and population-based Pharmaco-Epidemiological Prescription Database in North Jutland | Not specified | Cases: 55 (from a cohort of 26,005 patients treated with antidepressants)  Population of North Jutland county, Denmark: 490,000 | Users of antidepressants, aged 16–105 years, identified in the Pharmaco-Epidemiological Prescription Database during the period 1991–1995 and linked to the HDR | Cases: patients with a first admission to hospital with UGIB | *Range, n (%)* ≤ 59:  13,465 (52) 60–69:  4,471 (17) ≥ 70:  8,069 (31) | Current use: period of exposure, lasting from the date of prescription to 90 days after | Non-users: period of no exposure, extending from 90 days after prescription to date of next prescription | ‘Low-dose aspirin’ (not defined) | UGIB |
| de Abajo *et al*. (2001) [7] | Nested case–control (UK) | GPRD | *Random sample of 100 cases*: secondary prevention of CHD (n = 47), secondary prevention of cerebrovascular ischemic events (n = 36), PVD (n = 5), other CVD (n = 2), analgesia (n = 2), unknown (n = 8) | Cases (total): 2,105  UGIB: 1,833  Perforation: 272  Controls: 11,500 | Patients aged 40–79 years between April 1993 and October 1998, with at least 2 years' enrolment with their PCP | Cases: patients with an incident diagnosis for UGIB or perforation, aged 40–79 years, registered in the database  Controls: patients frequency-matched for age (interval 1 year), sex, and calendar year | *Range, n (%)*  (cases/controls) 40–59:  667 (31.7)/3,766 (32.8) 60–69:  627 (29.8)/3,392 (29.5) 70–79:  811 (38.5)/4,342 (37.8) | Current use: the supply of a prescription for aspirin lasted until the index date or ended within the period of 30 days before date of diagnosis | Non-users: if no prescription was ever recorded before the index date | 75–600+ mg Analysis by daily dose (as instructed): 75, 150, 300, and > 600 mg | UGIB |
| de Abajo *et al*. (2013) [8] | Nested case–control (Spain) | BIFAP (computerized, population-based, medical records database in Spain) | Cardioprotection; other indications not specified | Cases: 1,193  Controls: 10,000  (from a cohort of 669,115) | Persons aged 40–89 years in the period 2001–2005 who, at start date, did not have a record of cancer, esophageal varices, Mallory–Weiss syndrome, coagulopathies, alcohol-related disorders, and liver diseases | Cases: incident UGIB  Controls: randomly selected; frequency-matched for age, sex, and calendar year | Cases:  65.0 ± 13.2  Controls:  64.3 ± 13.2  *Range, n (%)*  (cases/controls)  40–54:  319 (26.7)/2,781 (27.8) 55–64:  238 (20.0)/2,093 (20.9) 65–74:  288 (24.1)/2,464 (24.6) 75+:  348 (29.2)/2,662 (26.6) | Current users: prescription lasted until index date or ended within 30 days prior to the index date | Non-users: no prescription or most recent prescription was > 365 days before index date | ≤ 300 mg/day | UGIB |
| De Berardis *et al*. (2012) [9] | Cohort, population-based (Italy) | Record-linkage of hospital discharge records, prescription databases, civil registries across 12 local health authorities in Puglia | Not specified | Low-dose aspirin: 186,425 Comparison cohort (not treated with low-dose aspirin): 186,425.  Cases: 6,907 (4,487 GI and 2,464 ICH) | New users of low-dose aspirin who were at least 30 years old, during the index period from January 2003 to December 2008, and had no aspirin prescriptions in the last year; and a comparison cohort of all those individuals who did not receive aspirin throughout the study period | Cases: patients hospitalized for a major GI bleeding or cerebral hemorrhage occurring after the initiation of antiplatelet therapy | Aspirin:  69.76 ± 11.29 Controls:  68.99 ± 11.83 | Current users: last prescription of aspirin at least 75 days before hospitaliza-tion for major bleeding events or end of follow-up | Non-users: individuals who did not receive aspirin throughout the study period | ≤ 300 mg/day | GI bleeding  ICH |
| Garcia-Rodriguez *et al*. (2011) [10] | Nested case–control within a cohort (UK) | THIN | Not specified | Cases: 2,049  Controls: 20,000 | All individuals recorded in THIN aged 40–84 years from 1 January 2000 to 31 December 2007, enrolled with their PCP for at least 2 years, and had a computerized prescription history of at least 1 year | Cases: patients with a recorded diagnosis of UGIB during the follow-up  Controls: individuals, frequency-matched by age, sex, and calendar year to the cases, which were randomly sampled from the same source population | Not specified | Current use: latest prescription lasting until the index date or ending within 30 days before the index date  Aspirin monotherapy was defined as current aspirin use with no use of the other antiplatelet drug in the previous year | Non-users: no exposure in the year before the index date | 75–300 mg/day | UGIB |
| Garcia-Rodriguez *et al*. (2013) [11] | Nested case–control within a cohort (UK) | THIN | Not specified | Cases (total): 3,137  ICH: 1,797  SAH: 1,340  Controls: 10,000  (from a cohort of 2,110,327) | All patients in the THIN database aged 20–89 years between January 2000 and December 2008  Patients with a diagnosis of ICH or SAH before their study start date were excluded from the study cohort | Cases: patients with an incident recorded diagnosis of hemorrhagic stroke  Controls: patients randomly selected from the pool of eligible person-time and frequency-matched to all cases for sex, age (± 1 year), and calendar year of diagnosis | ICH: 70.8 SAH: 57.7 | Current use: patients taking the drug at the index date or had taken it in the previous 30 days | Non-users: everyone else apart from current users | 75, 150 and 300 mg/day, all analyzed separately | ICH |
| Hallas *et al*. (2006) [12] | Case–control (Denmark) | Three Danish databases | Not specified | Cases: 1,443 Controls: 57,720 | Patients who had a hospital admission with peptic ulcer or gastritis with significant bleeding and potential bleeding source in the stomach or duodenum | Cases: defined by: admission with peptic ulcer or gastritis as the main diagnosis during 1 January 2000 to 31 December 2004; significant bleeding (melena, subnormal hemoglobin, or the need for transfusions); and a potential bleeding source in stomach or duodenum, identified by endoscopy or surgery  Controls: from general Danish population, matched for sex and age | Cases: 72.4 ± 14.3 Controls: 71.8 ± 14.3 | Current users: prescription in the previous 90 days  Recent users: prescription 91–180 days before index date  Past users: prescription > 180 days before index date | Non-users: currently non-exposed to all four classes of antithrombotic drugs (aspirin, clopidogrel, dipyridamole, and vitamin K antagonists) | ‘Low-dose aspirin’ (not defined) | UGIB |
| Hasan *et al*. (2011) [13] | Nested case–control within a cohort (USA/Canada/Europe) | ISUIA | Not specified | Total: 271 Cases: 58 Controls: 213 | Patients with at least one UIA; cases for this report were those who were in the initially untreated cohort and subsequently had a primary aneurysmal hemorrhage over a 5-year period after UIA diagnosis | Cases: patients enrolled in ISUIA who subsequently had a proven aneurysmal subarachnoid hemorrhage during a 5-year follow-up period.  Controls: matched to each case by site (anterior or posterior) and size (± 2 mm) of the UIA (4:1) | Overall: 57 | Aspirin use categories: never, less than once a month, once a month to 2 times a week, and 3 times a week to daily | No history of aspirin use | Analysis by frequency of use; dose and regimen not specified further | ICH |
| Hirata *et al*. (2011) [14] | Cohort, retrospective (Japan) | Medical records from a single hospital | Acute coronary syndrome (74.5%), atrial fibrillation (15.7%), or peripheral arterial disease (2.3%) | Low-dose aspirin  Total: 701 Buffered: 267 Enteric-coated: 434 Controls: 701 | Patients in the Division of Cardiovascular Disease who had been prescribed low-dose aspirin for CV disease for at least 1 year | Cases: patients receiving long-term low-dose aspirin for cardiovascular diseases  Controls: ambulatory patients who had not been prescribed low-dose aspirin and had a diagnosis of hypertension, in the same follow-up period as low-dose aspirin patients, and matched for age and sex | Cases: 73.1 ± 9.0 Control: 72.8 ± 9.1 | Not specified | Non-users (patients not prescribed low-dose aspirin) | ‘Low-dose aspirin’ (not defined) | GI bleeding  UGIB  LGIB |
| Ho *et al*. (2015) [15] | Cohort (China/Hong Kong) | Hospitalization records, laboratory and imaging results | Stroke prevention in patients with AF | Total: 8,754 | Chinese patients with atrial fibrillation and CHA2DS2-VASc ≥ 1 (CHA2DS2-VASc, 4.1 ± 1.5; HAS-BLED, 2.2 ± 0.9)  Therapy: warfarin, 16.3%; aspirin, 41.1%; dabigatran, 4.5%; no therapy, 38.1% | Patients who experienced ICH (intracerebral, subarachnoid, or subdural hemorrhage) | 79.5 ± 9.2 | Not specified | Patients receiving no antithrombotic therapy (38.1%) | 80–160 mg daily | ICH |
| Huang *et al*. (2010) [16] | Cohort, prospective (USA) | HPFS | Subset of 211 patients: CVD (25.4%), to decrease risk for CVD (58.4%), headaches (25.4%), joint or musculoskeletal pain (33.0%), and other reasons (7.0%) | Cases: 707  (from a cohort of 32,989 men) | Men enrolled in the HPFS in 1986 (aged 40–75 at baseline) who returned the 1994 questionnaire about aspirin use (excluding those with a prior history of GI bleeding, cancer, or peptic ulcer disease) | Cases: men reported an episode of major GI bleeding, between 2006 and 2008 | *By number of tablets (325 mg) per week* Non-use:  59.9 ± 9.3 0.5–1.5:  60.1 ± 9.1 2–5:  61.8 ± 9.2 6–14:  63.0 ± 9.1 > 14:  61.7 ± 8.8 | Regular users: ≥ 2 x 325 mg tablets per week | Non-users (no use of aspirin) or non-regular users (< 2 x 325 mg tablets per week) | Analysis by tablets (325 mg) per week:  0.5–1.5, 2–5, 6–14, and > 14 | GI bleeding  UGIB  LGIB |
| Huang *et al*. (2011) [17] | Cohort, prospective (USA) | Nurses' Health Study (NHS) | Subsample from cohort taking 1–6 aspirin/week: headache (32%), arthritis/musculo-skeletal pain (46%), CVD prevention (9%) and other (13%) | Cases: 1,537  (from a cohort of 87,680 women) | US female registered nurses enrolled in the Nurses’ Health Study in 1990 who provided biennial data on aspirin use, aged 30–55 years in the 1976 NHS | Cases: women who reported a major GI bleeding, during a 24-year follow-up (from 1990 to 2004) | *By number of tablets (325 mg) per week* Non-use:  56.5 ± 7.3 0.5–1.5:  55.9 ± 7.1 2–5: 56.8 ± 7.1 6–14:  57.8 ± 7.0 > 14:  58.8 ± 6.9 | Regular users: ≥ 2 x 325 mg tablets per week | Non-users (no use of aspirin) or non-regular users (< 2 x 325 mg tablets per week) | Analysis by tablets (325 mg) per week:  0.5–1.5, 2–5, 6–14, and > 14 | GI bleeding  UGIB  LGIB |
| Ibanez *et al*. (2006) [18] | Case–control, population-based (Spain/Italy) | Medical records from 18 hospitals | Not specified | Cases: 2,813  Controls: 7,193 | Patients recruited from September 1998 to December 2001 from 10 hospitals in Spain, and from November 1999 to December 2001 from eight hospitals in Italy | Cases: patients > 18 years admitted to hospital with a primary diagnosis of acute UGIB  Controls: patients admitted with non-alcohol-related trauma, elective surgery for non-painful disorders, and acute clinical conditions thought to be unrelated to intake of drugs of interest  Up to three controls per case were matched according to center, date of admission (± 2 months), sex, and age (± 5 years) | Not reported | Drug exposure: any use in the 7 days before the index day | Non-users: non-exposed individuals | Four categories: ≤ 100 , 101–150, 151–200, and > 200 mg/day | UGIB |
| Kaufman *et al*. (1993) [19] | Case–control (USA/Sweden/Hungary) | Medical records from 48 hospitals in eastern Massachusetts, Stockholm, and Budapest | Not specified | *Gastric/ duodenal bleeding* Cases:  335/239 Controls: 670/489 | – | Cases: patients with a first episode of major UGIB admitted to hospital  Controls:  USA (2:1): neighbors selected from town census  Sweden (3:1): neighbors selected from computerized population register  Hungary (2:1): hospital patients selected to have diagnoses unrelated to NSAID use | *Median*  Gastric bleeding: 62 Duodenal bleeding: 60 | Use: in the week before the event  Further subdivided into: regular users – at least every other day;  occasional users – less than regular, not specified further | Non-users: no use during the week before the index date | Dose categories: ≤ 250, 251–325, 326–999, 1000–1949, and ≥ 1950 mg | UGIB |
| Kaufman *et al*. (1999) [20] | Case–control (USA/Sweden) | USA: from Massachusetts Health Data Consortium, Inc. Sweden: from the Swedish register on hospital discharge diagnoses | Cardiovascular prophylaxis (≤ 325 mg/day dose) | Cases: 1,224 Controls: 2,945 | – | Cases: patients aged 18–79 years (no upper limit for Sweden), admitted with a diagnosis that might be related to acute UGIB due to gastric or duodenal ulcer or to gastritis (1987–1996 in USA and 1989–1992 in Sweden)  Controls: neighbors identified from town census lists (USA), and from computerized population registers (Sweden), matched to the cases (USA 2:1/Sweden 3:1), according to sex and half-decade of age | *Median* Cases: 63  Controls: 63 | Use: in the week before the event  Further subdivided into: regular users – at least every other day; occasional users – less than regular, not specified further | Non-users: likely no use during the week before the index date | Categories: > 325 and ≤ 325 mg | UGIB |
| Kelly *et al*. (1996) [21] | Case–control (USA) | Multicenter study conducted in 28 Massachusetts hospitals | CVD prevention among aspirin users (cases/controls): 21%/24% buffered, 20%/35% plain, and 37%/54% coated | Cases: 550 Controls: 1,202 | – | Cases: patients admitted to hospital with a first episode of major UGIB due to gastric or duodenal ulcer or gastritis  Controls: community controls identified from town census lists and matching cases by residence, sex, and half-decade of age (2:1) | *Median*  Gastric bleeding: 61 Duodenal bleeding: 60 Both: 55 Controls: 60 | Use: in the week before the event  Further subdivided into: regular users – at least every other day; occasional users – less than regular, not specified further | Non-users (not further defined) | 0 to > 325 mg/day  Categories: > 325 and ≤ 325 mg | UGIB |
| Lanas *et al*. (2000) [22] | Case–control, prospective (Spain) | Medical records from four general hospitals | Prophylaxis against vascular occlusive diseases | Cases: 1,122  Controls: 2,231 | – | Cases: patients hospitalized in one of the four participating centers with acute GI bleeding  Controls (2:1): selected from the same participating hospital and one from the community and matched with cases by sex and age (± 5 years) | Cases:  65.1 ± 16.6 Controls:  65.2 ± 16.7 | Current use: any time in 7 days before hospital admission or the day of the interview (for outpatient controls) | Not defined | ≤ 300 mg/day | UGIB |
| Lanas *et al*. (2006) [23] | Case–control, hospital-based (Spain) | Network of general hospitals integrated in the Spanish Association of Gastroenter-ology | Cardioprotection; other indications not specified | Cases: 2,777  Controls: 5,532 | Participants were 20–85 years old and were identified from a Spanish hospital network  They had been free of liver disease, coagulation disorders or malignancies during the previous 5 years | Cases: patients hospitalized due to GI bleeding  Controls (2:1): matched by age (difference of 5 years), hospital, and month of admission were selected | 61  *Range, %*  > 50: 75 | Current use: drug was taken up to 7 days before the index date  Current use of NSAIDs was further subdivided into single, switching, and multiple use | Non-users (not further defined) | Low-dose cardioprotective aspirin: ≤ 300 mg/day | UGIB |
| Lanas *et al*. (2015) [24] | Case–control (Spain) | Interviews by gastroenter-ologists, questionnaires about marketed drugs, reviews of prescriptions | Not specified | Cases: 1,008  Controls: 1,008 | Patients hospitalized for GI bleeding in general hospitals in the Spanish Association of Gastroenterology and the Biomedical Investigation Network Center of hepatic and digestive diseases | Cases: patients hospitalized for GI bleeding  Controls: matched by age (± 5 years), gender, hospital, and month of admission  Controls were obtained from people accompanying or visiting hospitalized patients, or referred to outpatient laboratory office for blood extraction | Cases:  66.6 ± 16  Controls:  65.6 ± 15.5 | Current use: taken in 7 days or less of hospitaliza-tion  Past use: when drug use ended more than 1 week before hospitaliza-tion | Non-users: individuals not reporting use | ≤ 300 mg/day | GI bleeding  UGIB  LGIB |
| Leung *et al*. (2009) [25] | Cohort (Hong Kong) | Hong Kong Diabetes Registry | Primary and secondary prevention of CVD | Total: 6,454  Primary/second-ary prevention: 5,731/723 | Chinese patients with type 2 diabetes, aged 30 years or older (between 1995 and 30 July 2005) | Cases: patients with UGIB and hemorrhagic stroke (‘secondary endpoints’) | Total:  58.0 ± 12.8  Aspirin users:  65.7 ± 10.2  Non-users:  55.4 ± 12.6 | Any use during the follow-up | Non-users: during the follow-up | 75–325 mg/day | UGIB  ICH |
| Lin *et al*. (2014) [26] | Nested case–control within a cohort (UK) | THIN | Primary and secondary prevention of CVD | Cases: 2,049 Controls 20,000  Primary prevention: 1,303 cases; 15,530 controls  Secondary prevention: 746 cases; 4,470 controls | Patients aged 40–84 years, who had been enrolled with their PCP for at least 2 years and who had at least 1 year of computerized prescription history, including a primary prevention cohort (no history of CVD) and a secondary prevention cohort (with history) | Cases: patients with a recorded diagnosis of UGIB  Controls: patients randomly selected from the pool of eligible person-time (i.e. using density-based sampling) and frequency-matched to all cases for sex, age, and calendar year of diagnosis | *Range, n*  (cases/controls)  Primary/  Secondary 40–69:  763/9,089  40–69:  240/1,146 70–79:  357/4,431  70–79:  355/2,156 80–84:  183/2,010  80–84:  151/1,168 | Current use: use lasting until the index date or ending in the 30 days before the index date  Monotherapy with low-dose aspirin was defined as current use of the drug in question with no use of the other antiplatelet drug in the previous year | Non-users: no exposure to the drug in the year before the index date | 75–300 mg/day | UGIB |
| Mousavi *et al*. (2013) [27] | Cohort, prospective (Iran) | Medical records from a single tertiary referral hospital | Not specified | Total: 633  Low-dose aspirin: 168  Non-use: 495 | Patients with GI hemorrhage who were admitted to hospital for endoscopy assessment | Cases: patients with GI hemorrhage who were admitted to hospital for endoscopy assessment | Low-dose aspirin:  62.49 ± 17.04 Non-use:  53.86 ± 19.54 | Low-dose aspirin 80–100 mg daily | Non-users (not further defined) | 80–100 mg/day | GI bleeding |
| Nakayama *et al*. (2009) [28] | Cohort, prospective (Japan) | Medical records from a single hospital | Not specified | Total: 285 | Patients with UGIB confirmed by endoscopy who were admitted to a single hospital in Japan, between January 2000 and December 2007 | Cases: bleeding ulcer patients who received emergency endoscopy in Saga medical school | 61.9 *Median*  62.0 | Daily use: regular intake over 4 weeks before endoscopy  On-demand use: at least 1 dose in 4 weeks before endoscopy | Non-users of aspirin | ≤ 300 mg/day | UGIB |
| Pilotto *et al*. (2003) [29] | Cohort, retrospective (Italy) | Medical records from the geriatric department of a single center | Low-dose aspirin (< 300 mg/day) ‘usually taken for prophylaxis of cardiovascular diseases’ | Cases 255  (from a cohort of 2,251) | Older patients (aged > 65 years) admitted consecutively to the geriatric department from January 1997 to December 2000, and who had undergone upper GI endoscopy | Cases: patients with endoscopically diagnosed upper GI tract bleeding | 80.18 ± 6.74  *Range*  65–102 | Users: patients who took an NSAID or aspirin at any time in the 7 days before endoscopy | Non-users of NSAIDs (not further defined) | ≤ 300 mg/day (low-dose) and > 300 mg/day (regular dose) | UGIB |
| Sakamoto *et al*. (2006) [30] | Case–control (Japan) | Medical records from 14 hospitals and population registries (for controls) | Not specified | Cases: 175 Controls: 347 | Patients aged ≤ 40 years who were admitted to the hospital due to a first episode of nonfatal major UGIB with hematemesis or melena  Duodenal ulcer, gastric ulcer, or gastritis were confirmed as the source of bleeding by endoscopy or at operation | Cases: patients with UGIB due to duodenal or gastric ulcer or gastritis  Controls: matched by age and sex were randomly selected from the population registries | *Median*: 60 *Range, n (%)*  (cases/controls) 40–49:  31 (18)/58 (17) 50–59:  53 (30)/112 (32) 60–69:  50 (29)/94 (27) 70–79:  29 (17)/63 (18) ≥ 80:  12 (7)/20 (6) | Users: patients who took a drug in 4 weeks before date of admission or interview | Non-users | Subdivided into < 325 and ≥ 325 mg/day | UGIB |
| Santolaria *et al*. (1999) [31] | Case–control, prospective (Spain) | Medical records from a single center | Vascular occlusive diseases (low-dose aspirin users) | *Bleeding/ uncomplicated ulcers* Cases: 185/75  Controls: 185/75 | – | Cases: patients consecutively attending the University Hospital of Zaragoza with bleeding peptic ulcers  Controls: consecutive patients admitted to the hospital for reasons that would not influence NSAID use, matched by age (± 5 years) and sex (controls for patients with uncomplicated PU were non-hospital patients) | *Patients with bleeding ulcers* Peptic:  54.9 ± 14.3 Duodenal:  53.1 ± 14.4 Gastric:  58.6 ± 12.7 Controls:  54.9 ± 15.2 | Users: if the drug had been taken in 1 week before date of admission for endoscopic diagnostic of peptic ulcer | Non-users | Low-dose: < 300 mg/day on a regular basis | UGIB |
| Sorensen *et al*. (2000) [32] | Cohort, retrospective, population-based (Denmark) | HDR and Pharmaco-Epidemiological Prescription Database in North Jutland | Cardioprotection; other indications not specified | Cases: 2,475 (from a cohort of 27,694)  Population of North Jutland county, Denmark: 490,000 | Users of low-dose (100 mg or 150 mg) aspirin tablets, aged 16–105 years, identified in the population-based Pharmaco-Epidemiological Prescription Database during 1991–1995 and linked to the HDR | Cases: patients with a first admission to hospital with UGIB | *Range, n (% total cohort)* Women/men 16–59:  1,979 (7)/2,980 (11) 60–69:  3,158 (11)/3,916 (14) ≥ 70:  8,728 (32)/6,923 (25) | Current use: period of exposure to low-dose aspirin, lasting from the date of prescription to 90 days after (or a censoring date) | Non-users: period of no exposure to aspirin extending from 90 days after prescription to date of next prescription | 100 mg/day (19.2%) or 150 mg/day (80.8%) | UGIB |
| Sostres *et al*. (2015) [33] | Case–control (Spain) | Interviews by gastroenter-ologists, questionnaires about marketed drugs, reviews of prescriptions | Not specified | Cases: 666  Controls: 666 | Patients hospitalized for GI bleeding in three general hospitals of  the Spanish National Health System | Cases: patients hospitalized for GI bleeding  Controls: matched by age ( ± 5 years), gender, hospital, and month of admission  Controls were obtained from people accompanying or visiting hospitalized patients, or referred to outpatient laboratory office for blood extraction | Cases:  61.6 ± 16.1  Controls:  60.4 ± 15.6 | Current use: taken in 7 days of hospitalization  Past use: when drug use ended more than 1 week before hospitaliza-tion | Non-users: individuals not reporting use | ≤ 300 mg/day | UGIB |
| Stack *et al*. (2002) [34] | Case–control (UK) | Medical records from a single hospital | Cardiovascular prophylaxis | Cases: 203  Controls:203 | – | Cases: patients admitted to the University and City Hospitals, Nottingham, for acute UGIB, over a 24-month period where the ulcer (gastric, duodenal, or both) was found at endoscopy  Controls (1:1): the next patient of the same sex and of similar age (± 5 years) admitted to the medical wards, within a maximum of 3 months of the index case admission | Cases:  66.0 ± 16.5 Controls:  66.5 ± 15.3 | Current use: any use within the week prior to hospital admission for UGIB | Not specified | 75 mg to > 300 mg  Low-dose aspirin defined as ≤ 300 mg/day | UGIB |
| Strate *et al*. (2011) [35] | Cohort, prospective (USA) | HPFS | Subset of 211 patients: CVD (25.4%), to decrease risk for CVD (58.4%), headaches (25.4%), joint or musculoskeletal pain (33.0%), and other reasons (7.0%) | Cases: 1,195 cases from a cohort of 47,210 men (939 cases of diverticulitis and 256 cases of diverticular bleeding) | Men enrolled in the HPFS who were aged 40–75 years in 1986 and returned a detailed medical and dietary questionnaire | Cases: diverticular bleeding based on responses to biennial (from 1990 to 2006) and supplemental questionnaires (from 2006 to 2008) | Regular users:  56 ± 9.7 Non-regular users:  53 ± 9.7 | Regular use of aspirin only, NSAIDs only, or aspirin and NSAIDs: 2 or more times/week | Non-regular users of aspirin and NSAIDs: fewer than 2 times/week  Non-users of aspirin or NSAIDs:  men who denied use of these medications  Non-users of aspirin:  men who denied use of aspirin) | From 0.1 to ≥ 6 x 325 mg tablets/week | LGIB |
| Taha *et al*. (2006) [36] | Cohort, retrospective (UK) | Medical records from a single center | Not specified | Total: 674 | Patients with hematemesis and/or melena and presenting to a single hospital, over a total period of three complete years: 1996, 1999, and 2002 | Patients with hematemesis and/or melena and presenting to Crosshouse Hospital, Glasgow over a total period of 3 complete years: 1996, 1999, and 2002 | *Median (IQR)*  60 (44–77) | Not specified | Non-users of aspirin | 75 mg/day | UGIB |
| Thrift *et al*. (1999) [37] | Case–control (Australia) | Medical records from 13 hospitals | Not specified | Cases: 331 Controls: 331 | Consecutive cases of primary intracerebral hemorrhage | Cases: consecutive cases of primary intracerebral hemorrhage verified by computed tomography or post-mortem examination  Control: matched by age, sex, and socioeconomic status (identified as individuals living on the same street in which the case lived at the time of event) | Cases:  63.4 ± 12.4 Controls:  63.4 ± 12.4 | Only use over the 14 days preceding the event (or interview) was recorded | Non-users in the 14-day period | 100–175 mg/day (low-dose) | ICH |
| Wu *et al*. (2014) [38] | Case-crossover (Taiwan) | A database randomly sampled from Taiwan's national health insurance database | Not specified | 10,905 cases from a cohort of 501,946 (7,124 GI and 3,781 cerebral hemorrhage)  Of these, 489 used low-dose aspirin in the case-only period and 294 in the control only period | Han Chinese patients, aged 30–95 years old, at risk of a major bleeding event, randomly selected from those registered in NHI database in 2000 | Cases: patients hospitalized for a primary diagnosis of incident hemorrhagic complications and prescribed low-dose aspirin (≤ 300 mg/day) from 2000to 2008 | *Range, n (%)* 30–64:  5,528 (50.7) ≥ 65:  5,377 (49.3) | Use in 56-day case period only: defined as 1–56 days before first diagnosis of major hemorrhagic complication (and, by definition, non-use during the 56-day control period [57–112 days before the index date]) | Users during the control period  Non-users during the case period | ≤ 300 mg/day | GI bleeding  ICH |
| Yamada *et al*. (2008) [39] | Case–control (Japan) | Medical records from three hospitals | Not specified | Cases: 44 Controls: 88  (from a total of 9,499 patients undergoing colonoscopy) | All colonoscopies performed between September 1995 and December 2005 at the three hospitals | Cases: patients with recent hematochezia, for which urgent colonoscopy was indicated on hospitalization and performed within 4 days after the episode, identified from September 1995 and December 2005  Controls (2:1): patients with colonic diverticulosis without history of bleeding and matching age (± 2 years), gender, and location of diverticulosis | Cases:  67.2 ± 12.8 (not specified for controls) | Not specified | Not specified | ≤ 100 mg/day | LGIB |

ACG, American College of Gastroenterology; APT, Alimentary Pharmacology and therapeutics; BIFAP, Base de datos para la Investigación Farmacoepidemiológica en Atención Primari; CVD, cardiovascular disease; GI, gastrointestinal; GPRD, (UK-based) General Practice Research Database; HDR, Hospital Discharge Register; HPFS, Health Professionals Follow-up study; ICD, International Classification of Diseases; ICH, intracranial hemorrhage; IQR, interquartile range; ISUIA, International Study of Unruptured Intracranial Aneurysms; LGIB, lower gastrointestinal bleeding; NHI, National Health Insurance; NHS, National Health Service; NSAID, non-steroidal anti-inflammatory drug; PCP, primary care physician; SD, standard deviation; THIN, The UK Health Improvement Network; UGIB, upper gastrointestinal bleeding; UIA, unruptured intracranial aneurysm; WHS, Women’s Health Study.

References

1. Ahsberg K, Hoglund P, Stael von Holstein C. Mortality from peptic ulcer bleeding: the impact of comorbidity and the use of drugs that promote bleeding. Aliment Pharmacol Ther. 2010;32(6):801-10.

2. Ahsberg K, Hoglund P, Kim WH, von Holstein CS. Impact of aspirin, NSAIDs, warfarin, corticosteroids and SSRIs on the site and outcome of non-variceal upper and lower gastrointestinal bleeding. Scand J Gastroenterol. 2010;45(12):1404-15.

3. Blot WJ, McLaughlin JK. Over the counter non-steroidal anti-inflammatory drugs and risk of gastrointestinal bleeding. Journal of epidemiology and biostatistics. 2000;5 (2):137-42.

4. Cea Soriano L, Rodriguez LA. Risk of Upper Gastrointestinal Bleeding in a Cohort of New Users of Low-Dose ASA for Secondary Prevention of Cardiovascular Outcomes. Front Pharmacol. 2010;1:126.

5. Cook NR, Lee IM, Zhang SM, Moorthy MV, Buring JE. Alternate-day, low-dose aspirin and cancer risk: Long-term observational follow-up of a randomized trial. Annals of Internal Medicine. 2013;159 (2):77-85.

6. Dalton SO, Johansen C, Mellemkjaer L, Norgard B, Sorensen HT, Olsen JH. Use of selective serotonin reuptake inhibitors and risk of upper gastrointestinal tract bleeding: a population-based cohort study. Arch Intern Med. 2003;163(1):59-64.

7. de Abajo FJ, Garcia Rodriguez LA. Risk of upper gastrointestinal bleeding and perforation associated with low-dose aspirin as plain and enteric-coated formulations. BMC Clinical Pharmacology. 2001;1(1).

8. de Abajo FJ, Gil MJ, Bryant V, Timoner J, Oliva B, Garcia-Rodriguez LA. Upper gastrointestinal bleeding associated with NSAIDs, other drugs and interactions: A nested case-control study in a new general practice database. European Journal of Clinical Pharmacology. 2013;69 (3):691-701.

9. De Berardis G, Lucisano G, D'Ettorre A, Pellegrini F, Lepore V, Tognoni G, et al. Association of aspirin use with major bleeding in patients with and without diabetes. JAMA. 2012;307(21):2286-94.

10. Garcia Rodriguez LA, Lin KJ, Hernandez-Diaz S, Johansson S. Risk of upper gastrointestinal bleeding with low-dose acetylsalicylic acid alone and in combination with clopidogrel and other medications. Circulation. 2011;123 (10):1108-15.

11. Garcia-Rodriguez LA, Gaist D, Morton J, Cookson C, Gonzalez-Perez A. Antithrombotic drugs and risk of hemorrhagic stroke in the general population. Neurology. 2013;81(6):566-74.

12. Hallas J, Dall M, Andries A, Andersen BS, Aalykke C, Hansen JM, et al. Use of single and combined antithrombotic therapy and risk of serious upper gastrointestinal bleeding: Population based case-control study. British Medical Journal. 2006;333 (7571):726-8.

13. Hasan DM, Mahaney KB, Brown RD, Jr., Meissner I, Piepgras DG, Huston J, et al. Aspirin as a promising agent for decreasing incidence of cerebral aneurysm rupture. Stroke. 2011;42(11):3156-62.

14. Hirata Y, Kataoka H, Shimura T, Mizushima T, Mizoshita T, Tanida S, et al. Incidence of gastrointestinal bleeding in patients with cardiovascular disease: Buffered aspirin versus enteric-coated aspirin. Scandinavian Journal of Gastroenterology. 2011;46 (7-8):803-9.

15. Ho CW, Ho MH, Chan PH, Hai JJ, Cheung E, Yeung CY, et al. Ischemic stroke and intracranial hemorrhage with aspirin, dabigatran, and warfarin: Impact of quality of anticoagulation control. Stroke. 2015;46 (1):23-30.

16. Huang ES, Strate LL, Ho WW, Lee SS, Chan AT. A prospective study of aspirin use and the risk of gastrointestinal bleeding in men. PLoS One. 2010;5(12):e15721.

17. Huang ES, Strate LL, Ho WW, Lee SS, Chan AT. Long-term use of aspirin and the risk of gastrointestinal bleeding. American Journal of Medicine. 2011;124 (5):426-33.

18. Ibanez L, Vidal X, Vendrell L, Moretti U, Laporte JR. Upper gastrointestinal bleeding associated with antiplatelet drugs. Aliment Pharmacol Ther. 2006;23(2):235-42.

19. Kaufman DW, Kelly JP, Sheehan JE, Laszlo A, Wiholm BE, Alfredsson L, et al. Nonsteroidal anti-inflammatory drug use in relation to major upper gastrointestinal bleeding. Clinical Pharmacology and Therapeutics. 1993;53 (4):485-94.

20. Kaufman DW, Kelly JP, Wiholm BE, Laszlo A, Sheehan JE, Koff RS, et al. The risk of acute major upper gastrointestinal bleeding among users of aspirin and ibuprofen at various levels of alcohol consumption. American Journal of Gastroenterology. 1999;94 (11):3189-96.

21. Kelly JP, Kaufman DW, Jurgelon JM, Sheehan J, Koff RS, Shapiro S. Risk of aspirin-associated major upper-gastrointestinal bleeding with enteric-coated or buffered product. Lancet. 1996;348 (9039):1413-6.

22. Lanas A, Bajador E, Serrano P, Fuentes J, Carreno S, Guardia J, et al. Nitrovasodilators, low-dose aspirin, other nonsteroidal antiinflammatory drugs, and the risk of upper gastrointestinal bleeding. N Engl J Med. 2000;343(12):834-9.

23. Lanas A, Garcia-Rodriguez LA, Arroyo MT, Gomollon F, Feu F, Gonzalez-Perez A, et al. Risk of upper gastrointestinal ulcer bleeding associated with selective cyclo-oxygenase-2 inhibitors, traditional non-aspirin non-steroidal anti-inflammatory drugs, aspirin and combinations. Gut. 2006;55 (12):1731-8.

24. Lanas A, Carrera-Lasfuentes P, Arguedas Y, Garcia S, Bujanda L, Calvet X, et al. Risk of Upper and Lower Gastrointestinal Bleeding in Patients Taking Nonsteroidal Anti-inflammatory Drugs, Antiplatelet Agents, or Anticoagulants. Clin Gastroenterol Hepatol. 2015;13(5):906-12 e2.

25. Leung WY, So WY, Stewart D, Lui A, Tong PC, Ko GT, et al. Lack of benefits for prevention of cardiovascular disease with aspirin therapy in type 2 diabetic patients - a longitudinal observational study. Cardiovascular Diabetology. 2009;8 (pp 57)(1475).

26. Lin KJ, De Caterina R, Garcia Rodriguez LA. Low-dose aspirin and upper gastrointestinal bleeding in primary versus secondary cardiovascular prevention a population-based, nested case-control study. Circulation: Cardiovascular Quality and Outcomes. 2014;7 (1):70-7.

27. Mousavi M, Salehimarzijarani B, Dadvar Z, Jalaly NY, Valizadeh N, Sotoudeh H, et al. Relationship between continuous use of low-dose enteric-coated aspirin and gastrointestinal injuries in patients with gastrointestinal hemorrhage. Turkish Journal of Gastroenterology. 2013;24 (2):93-8.

28. Nakayama M, Iwakiri R, Hara M, Ootani H, Shimoda R, Tsunada S, et al. Low-dose aspirin is a prominent cause of bleeding ulcers in patients who underwent emergency endoscopy. J Gastroenterol. 2009;44(9):912-8.

29. Pilotto A, Franceschi M, Leandro G, Paris F, Niro V, Longo MG, et al. The risk of upper gastrointestinal bleeding in elderly users of aspirin and other non-steroidal anti-inflammatory drugs: the role of gastroprotective drugs. Aging Clin Exp Res. 2003;15(6):494-9.

30. Sakamoto C, Sugano K, Ota S, Sakaki N, Takahashi S, Yoshida Y, et al. Case-control study on the association of upper gastrointestinal bleeding and nonsteroidal anti-inflammatory drugs in Japan. Eur J Clin Pharmacol. 2006;62(9):765-72.

31. Santolaria S, Lanas A, Benito R, Perez-Aisa M, Montoro M, Sainz R. Helicobacter pylori infection is a protective factor for bleeding gastric ulcers but not for bleeding duodenal ulcers in NSAID users. Aliment Pharmacol Ther. 1999;13(11):1511-8.

32. Sorensen HT, Mellemkjaer L, Blot WJ, Nielsen GL, Steffensen FH, McLaughlin JK, et al. Risk of upper gastrointestinal bleeding associated with use of low-dose aspirin. Am J Gastroenterol. 2000;95(9):2218-24.

33. Sostres C, Carrera-Lasfuentes P, Benito R, Roncales P, Arruebo M, Arroyo MT, et al. Peptic Ulcer Bleeding Risk. The Role of Helicobacter Pylori Infection in NSAID/Low-Dose Aspirin Users. American Journal of Gastroenterology. 2015;110(5):684-9.

34. Stack WA, Atherton JC, Hawkey GM, Logan RF, Hawkey CJ. Interactions between Helicobacter pylori and other risk factors for peptic ulcer bleeding. Aliment Pharmacol Ther. 2002;16(3):497-506.

35. Strate LL, Liu YL, Huang ES, Giovannucci EL, Chan AT. Use of aspirin or nonsteroidal anti-inflammatory drugs increases risk for diverticulitis and diverticular bleeding. Gastroenterology. 2011;140(5):1427-33.

36. Taha AS, Angerson WJ, Knill-Jones RP, Blatchford O. Upper gastrointestinal mucosal abnormalities and blood loss complicating low-dose aspirin and antithrombotic therapy. Aliment Pharmacol Ther. 2006;23(4):489-95.

37. Thrift AG, McNeil JJ, Forbes A, Donnan GA. Risk of primary intracerebral haemorrhage associated with aspirin and non-steroidal anti-inflammatory drugs: case-control study. BMJ. 1999;318(7186):759-64.

38. Wu IC, Hsieh HM, Yu FJ, Wu MC, Wu TS, Wu MT. A Long-term Risk-Benefit Analysis of Low-dose Aspirin in Primary Prevention. Eur J Clin Invest. 2015.

39. Yamada A, Sugimoto T, Kondo S, Ohta M, Watabe H, Maeda S, et al. Assessment of the risk factors for colonic diverticular hemorrhage. Dis Colon Rectum. 2008;51(1):116-20.
