# Supplementary material for: Bleeding Risk with Long-Term Low-Dose Aspirin: A Systematic Review of Observational Studies
Source: PLoS One. 2016 Aug 4;11(8):e0160046. doi: 10.1371/journal.pone.0160046 (PMC4973997; doi:10.1371/journal.pone.0160046)
Supplement: S2 Table — Values are adjusted or multivariate odds ratios unless otherwise indicated. aAdjusted RR. bNSAIDs included aspirin. CI, confidence interval; D, duodenal ulcer; G, gastric ulcer; UGIB, upper gastrointestinal bleeding. (DOCX) [file pone.0160046.s009.docx]

**S2 Table.** Effect of *Helicobacter* *pylori* infection on the risk of UGIB.

| Study | *H. pylori* measure | UGIB events, n (%) | | Odds ratio  (95% CI) |
| --- | --- | --- | --- | --- |
|  |  | Cases | Controls |  |
| Stack et al. (2002) [[44](#_ENREF_44)] | *H. pylori*-positive serology | 150 (73.9) | 109 (53.7) | – |
|  | *CagA*-positive | – (51.6) | – (28.5) | 3.3 (1.7–6.6) |
|  | *CagA*-negative | – (19.2) | – (21.0) | 1.6 (0.7–3.7) |
| Santolaria et al. (1999) [[43](#_ENREF_43)] | *H. pylori* infection |  |  |  |
|  | All participants | D: 114 (90.5)  G: 48 (81.4) | 123 (66.5) | D: 5.98 (2.9–12.3)  G: 1.7 (0.7–4.1) |
|  | Low-dose aspirin users | D: 16 (13)  G: 12 (20.7) | 11 (5.9) | D: 0.7 (0.1–4.6)  G: 0.6 (0.11–3.6) |
| Sostres et al. (2015) [[45](#_ENREF_45)] | *H. pylori* infection | 495 (74.3) | 365 (54.8) | 2.6 (2.0–3.3)^a^ |
|  | *H. pylori* infection + no low-dose aspirin | 425 | 321 | 2.8 (2.0–3.8) |
|  | No *H. Pylori* infection + low-dose aspirin | 35 | 36 | 2.2 (1.2–4.2) |
|  | *H. pylori* infection + low-dose aspirin | 70 | 44 | 3.5 (2.0–6.1) |
| Sakamoto et al. (2006) [[42](#_ENREF_42)] | *H. pylori* infection | 152 (86.9) | 143 (63.6) | 4.9 (2.4–10.2) |
|  | *H. pylori* infection + no NSAID^b^ | 114 | 122 | 5.4 (2.8-10.4) |
|  | No *H. Pylori* infection + NSAID^b^ | 11 | 13 | 4.9 (1.8-13.4) |
|  | *H. pylori* infection + NSAID^b^ | 38 | 21 | 10.4 (4.6--23.4) |
|  |  |  |  |  |
| Nakayama et al. (2009) [[54](#_ENREF_54)] | *H. pylori* infection | 221 (77.5) | – | – |
| Hallas et al. (2006) [[35](#_ENREF_35)] | History of *H. pylori* eradication | 96 (6.7) | 1123 (1.9) | – |

Values are adjusted or multivariate odds ratios unless otherwise indicated.

^a^Adjusted RR.

^b^NSAIDs included aspirin.

CI, confidence interval; D, duodenal ulcer; G, gastric ulcer; UGIB, upper gastrointestinal bleeding.
