# Supplementary material for: Bleeding Risk with Long-Term Low-Dose Aspirin: A Systematic Review of Observational Studies
Source: PLoS One. 2016 Aug 4;11(8):e0160046. doi: 10.1371/journal.pone.0160046 (PMC4973997; doi:10.1371/journal.pone.0160046)
Supplement: S3 Table — aIncidence rate ratio. CI, confidence interval; LGIB, lower gastrointestinal bleeding; OR, odds ratio; PPI, proton pump inhibitor; RR, relative risk; UGIB, upper gastrointestinal bleeding. (DOCX) [file pone.0160046.s010.docx]

**S3 Table.** Effect of proton pump inhibitor use on the risk of major bleeding events.

| Study | Outcome | Medication | Comparator | Adjusted OR/RR (95% CI) |
| --- | --- | --- | --- | --- |
| De Berardis et al. (2012) [[34](#_ENREF_34)] | Major bleeding | PPI | No PPI | 0.84 (0.80–0.88)^a^ |
|  |  | PPI + low-dose aspirin | PPI + no low-dose aspirin | 1.04 (0.96–1.12) |
| Cea Soriano et al. (2010) [[23](#_ENREF_23)] | UGIB | PPI (current) in low-dose aspirin users | No PPI (in low-dose aspirin users) | 0.97 (0.65–1.44) |
|  |  | PPI (current, initiated at start date) in low-dose aspirin users | No PPI (in low-dose aspirin users) | 0.56 (0.33–0.96) |
| Ibanez et al. (2006) [[36](#_ENREF_36)] | UGIB | PPI | No PPI | 0.6 (0.5–0.8) |
|  |  | Low-dose aspirin | No low-dose aspirin | 4.0 (3.2–4.9) |
|  |  | Low-dose aspirin + PPI | No low-dose aspirin | 1.1 (0.5–2.6) |
| Lanas et al. (2015) [[18](#_ENREF_18)] | UGIB | PPI | No PPI | 0.4 (0.3–0.5) |
|  |  | Low-dose aspirin + no PPI | No low-dose aspirin + no PPI | 1.99 (1.15–3.44) |
|  |  | No low-dose aspirin + PPI | No low-dose aspirin + no PPI | 0.42 (0.28–0.65) |
|  |  | Aspirin + PPI | No low-dose aspirin + no PPI | 0.62 (0.35–1.08) |
|  | LGIB | PPI | No PPI | 1.5 (1.1–1.9) |
| Pilotto et al. (2003) [[47](#_ENREF_47)] | UGIB | PPI | No PPI | 0.37 (0.18–0.75) |
|  |  | PPI + acute NSAID and/or aspirin | No PPI + no acute NSAID and/or aspirin | 1.05 (0.19–5.65) |
|  |  | PPI + chronic NSAID and/or aspirin | No PPI + no chronic NSAID and/or aspirin | 1.12 (0.21–6.07) |
| Lanas et al. (2000) [[40](#_ENREF_40)] | UGIB | Low-dose aspirin + omeprazole | Low-dose aspirin | 0.2 (0.1–0.9) |
|  |  |  |  |  |
|  |  |  |  |  |
|  |  |  |  |  |

^a^Incidence rate ratio.

^b^NSAIDs included aspirin.

CI, confidence interval; LGIB, lower gastrointestinal bleeding; OR, odds ratio; PPI, proton pump inhibitor; RR, relative risk; UGIB, upper gastrointestinal bleeding.
