# Supplementary material for: Bleeding Risk with Long-Term Low-Dose Aspirin: A Systematic Review of Observational Studies
Source: PLoS One. 2016 Aug 4;11(8):e0160046. doi: 10.1371/journal.pone.0160046 (PMC4973997; doi:10.1371/journal.pone.0160046)
Supplement: S4 Table — Values are adjusted or multivariate OR, RR, or HR unless otherwise indicated. aStandardized incidence ratio. bNo aspirin, no clopidogrel, no vitamin K antagonist, and no dipyridamole. cSecondary prevention cohort. dIncidence rate ratio. CI, confidence interval; coxib, cyclo-oxygenase 2 inhibitor; HR, hazard ratio; LGIB, lower gastrointestinal bleeding; OR, odds ratio; RR, relative risk; UGIB, upper gastrointestinal bleeding. (DOCX) [file pone.0160046.s011.docx]

**S4 Table.** Risk of major bleeding events with concomitant medications: clopidogrel, NSAIDs, and anticoagulants.

| Study | Outcome | Medication | Comparator | OR/RR/HR  (95% CI) |
| --- | --- | --- | --- | --- |
| *NSAIDs* |  |  |  |  |
| Cea Soriano et al. (2010) [[23](#_ENREF_23)] | UGIB | Low-dose aspirin + NSAID | Low-dose aspirin + no NSAID | 2.92 (1.77–4.82) |
| de Abajo et al. (2001) [[24](#_ENREF_24)] | UGIB/  perforation | Low-dose aspirin alone | No low-dose aspirin + no NSAID | 2.1 (1.8–2.5) |
|  |  | Low/medium dose NSAID alone | No low-dose aspirin + no NSAID | 2.6 (2.0–3.4) |
|  |  | High-dose NSAID alone | No low-dose aspirin + no NSAID | 4.3 (3.6–5.2) |
|  |  | Low-dose aspirin + low/medium dose NSAID | No low-dose aspirin + no NSAID | 2.2 (1.0–4.6) |
|  |  | Low-dose aspirin + high-dose NSAID | No low-dose aspirin + no NSAID | 13.3 (8.5–20.9) |
| de Abajo et al. (2013) [[25](#_ENREF_25)] | UGIB | Low-dose aspirin + NSAID | No low-dose aspirin + no NSAID | 2.99 (1.71–5.23) |
|  |  | Low-dose aspirin + NSAID | No low-dose aspirin + NSAID | 1.73 (0.97–3.08) |
| Garcia Rodriguez et al. (2011) [[26](#_ENREF_26)] | UGIB | Low-dose aspirin + low/medium dose NSAID | Low-dose aspirin + no NSAID | 2.63 (1.93–3.60) |
|  |  | Low-dose aspirin + high-dose NSAID | Low-dose aspirin + no NSAID | 2.66 (1.88–3.76) |
| Lanas et al. (2000) [[40](#_ENREF_40)] | UGIB | Low-dose aspirin + other NSAID | Low-dose aspirin + no NSAID | 3.8 (1.8–7.8) |
| Lanas et al. (2006) [[41](#_ENREF_41)] | UGIB | NSAID alone | No low-dose aspirin + no NSAID | 5.3 (4.4–6.3) |
|  |  | Low-dose aspirin alone | No low-dose aspirin + no NSAID | 3.9 (3.1–4.9) |
|  |  | NSAID + low-dose aspirin | No low-dose aspirin + no NSAID | 12.7 (7.0–23.0) |
|  |  | Coxib alone | No low-dose aspirin + no coxib | 1.0 (0.5–1.8) |
|  |  | Low-dose aspirin alone | No low-dose aspirin + no coxib | 3.6 (2.9–4.5) |
|  |  | Low-dose aspirin + coxib | No low-dose aspirin + no coxib | 14.5 (3.3–63.9) |
| Sorensen et al. (2000) [[48](#_ENREF_48)] | UGIB | Low-dose aspirin alone | No low-dose aspirin | 2.6 (2.2–2.9)^a^ |
|  |  | Low-dose aspirin + NSAID | No low-dose aspirin + no NSAID | 5.6 (4.4–7.0)^a^ |
| Strate et al. (2011) [[51](#_ENREF_51)] | LGIB | Low-dose aspirin alone | No low-dose aspirin + no NSAID | 1.70 (1.21–2.39) |
|  |  | NSAID alone | No low-dose aspirin + no NSAID | 1.74 (1.15–2.64) |
|  |  | Low-dose aspirin + NSAID | No low-dose aspirin + no NSAID | 2.02 (1.38–2.96) |
| *Clopidogrel* |  |  |  |  |
| Cea Soriano et al. (2010) [[23](#_ENREF_23)] | UGIB | Low-dose aspirin + clopidogrel | Low-dose aspirin + no clopidogrel | 1.61 (0.85–3.05) |
| Garcia Rodriguez et al. (2011) [[26](#_ENREF_26)] | UGIB | Low-dose aspirin + clopidogrel | Low-dose aspirin + no clopidogrel | 2.08 (1.34–3.21) |
| Hallas et al. (2006) [[35](#_ENREF_35)] | Serious UGIB | Low-dose aspirin alone | No antithrombotics^b^ | 1.8 (1.5–2.1) |
|  |  | Clopidogrel alone | No antithrombotics^b^ | 1.1 (0.6–2.1) |
|  |  | Low-dose aspirin + clopidogrel | No antithrombotics^b^ | 7.4 (3.5–15) |
| Lin et al. (2014) [[29](#_ENREF_29)]^c^ | UGIB | Low-dose aspirin alone | No low-dose aspirin + no clopidogrel | 1.40 (1.14–1.72) |
|  |  | Clopidogrel alone | No low-dose aspirin + no clopidogrel | 1.29 (0.82–2.03) |
|  |  | Low-dose aspirin + clopidogrel | No low-dose aspirin + no clopidogrel | 3.06 (1.92–4.89) |
| Garcia Rodriguez et al. (2013) [[27](#_ENREF_27)] | Intracerebral hemorrhage | Low-dose aspirin + no clopidogrel | No low-dose aspirin + no clopidogrel | 1.07 (0.94–1.23) |
|  |  | Low-dose aspirin + clopidogrel | No low-dose aspirin + no clopidogrel | 0.72 (0.36–1.44) |
|  | Subarachnoid hemorrhage | Low-dose aspirin + no clopidogrel | No low-dose aspirin + no clopidogrel | 0.81 (0.66–1.00) |
|  |  | Low-dose aspirin + clopidogrel | No low-dose aspirin + no clopidogrel | 0.90 (0.35–2.35) |
| *Other antiplatelet agents and/or anticoagulants* |  |  |  |  |
| De Berardis et al. (2012) [[34](#_ENREF_34)] | Major bleed | Low-dose aspirin + other antiplatelet | No low-dose aspirin + other antiplatelet | 2.56 (2.15–3.04)^d^ |
|  |  | Low-dose aspirin + other anticoagulants | No low-dose aspirin + other anticoagulants | 1.92 (1.65–2.24)^d^ |
|  |  | Low-dose aspirin + other antiplatelet + other anticoagulant | No low-dose aspirin + other antiplatelet + other anticoagulant | 2.89 (1.71–4.88)^d^ |
| de Abajo et al. (2001) [[24](#_ENREF_24)] | UGIB/  perforation | Low-dose aspirin alone | No low-dose aspirin + no NSAID | 2.1 (1.8–2.5) |
|  |  | Low-dose aspirin + anticoagulants | No antithrombotics^b^ | 5.5 (2.4–12.7) |
| Hallas et al. (2006) [[35](#_ENREF_35)] | Serious UGIB | Low-dose aspirin alone | No antithrombotics^b^ | 1.8 (1.5–2.1) |
|  |  | Vitamin K antagonist alone | No antithrombotics^b^ | 1.8 (1.3–2.4) |
|  |  | Dipyridamole alone | No antithrombotics^b^ | 1.9 (1.3–2.8) |
|  |  | Low-dose aspirin + vitamin K antagonist | No antithrombotics^b^ | 5.3 (2.9–9.5) |
|  |  | Low-dose aspirin + dipyridamole | No antithrombotics^b^ | 2.3 (1.7–3.3) |
| Garcia Rodriguez et al. (2013) [[27](#_ENREF_27)] | Intracerebral hemorrhage | Low-dose aspirin + no dipyridamole | No low-dose aspirin + no dipyridamole | 1.01 (0.88–1.16) |
|  |  | Low-dose aspirin + dipyridamole | No low-dose aspirin + no dipyridamole | 2.97 (1.80–4.90) |
|  | Subarachnoid hemorrhage | Low-dose aspirin + no dipyridamole | No low-dose aspirin + no dipyridamole | 0.80 (0.65–0.98) |
|  |  | Low-dose aspirin + dipyridamole | No low-dose aspirin + no dipyridamole | 2.37 (1.02–5.47) |

Values are adjusted or multivariate OR, RR, or HR unless otherwise indicated.

^a^Standardized incidence ratio.

^b^No aspirin, no clopidogrel, no vitamin K antagonist, and no dipyridamole.

^c^Secondary prevention cohort.

^d^Incidence rate ratio.

CI, confidence interval; coxib, cyclo-oxygenase 2 inhibitor; HR, hazard ratio; LGIB, lower gastrointestinal bleeding; OR, odds ratio; RR, relative risk; UGIB, upper gastrointestinal bleeding.
