# Supplementary material for: Bleeding Risk with Long-Term Low-Dose Aspirin: A Systematic Review of Observational Studies
Source: PLoS One. 2016 Aug 4;11(8):e0160046. doi: 10.1371/journal.pone.0160046 (PMC4973997; doi:10.1371/journal.pone.0160046)
Supplement: S5 Table — aRatio of observed risk to expected risk. bIncidence rate ratio. CI, confidence interval; coxib, cyclo-oxygenase 2 inhibitor; HR, hazard ratio; LGIB, lower gastrointestinal bleeding; OR, odds ratio; RR, relative risk; SSRI, selective serotonin reuptake inhibitor; UGIB, upper gastrointestinal bleeding. (DOCX) [file pone.0160046.s012.docx]

**S5 Table.** Effect of SSRI use on the risk of major bleeding events.

| Study | Outcome | Medication | Comparator | Adjusted RR (95% CI) |
| --- | --- | --- | --- | --- |
| Garcia Rodriguez et al. (2011) [[26](#_ENREF_26)] | UGIB | SSRI (current) | No SSRI | 1.20 (0.97–1.48) |
|  |  | SSRI (past) | No SSRI | 1.26 (0.96–1.65) |
|  |  | SSRI (current) +  low-dose aspirin | No SSRI +  low-dose aspirin | 0.80 (0.54–1.20) |
| Dalton et al. (2003) [[55](#_ENREF_55)] | UGIB | SSRI | No SSRI | 3.6 (2.7–4.7)^a^ |
|  |  | Low-dose aspirin alone | No low-dose aspirin + no SSRI | 2.5 (2.2–2.9)^a^ |
|  |  | Low-dose aspirin + SSRI | No low-dose aspirin + no SSRI | 5.2 (3.2–8.0)^a^ |
| De Berardis et al. (2012) [[34](#_ENREF_34)] | Major bleed | SSRI + low-dose aspirin | SSRI + no low-dose aspirin | 1.04 (0.96–1.12)^b^ |

^a^Ratio of observed risk to expected risk.

^b^Incidence rate ratio.

CI, confidence interval; coxib, cyclo-oxygenase 2 inhibitor; HR, hazard ratio; LGIB, lower gastrointestinal bleeding; OR, odds ratio; RR, relative risk; SSRI, selective serotonin reuptake inhibitor; UGIB, upper gastrointestinal bleeding.
